# A PRISMA systematic review through time on predictive musculoskeletal simulations

**DOI:** 10.1186/s12984-025-01686-w

**Published:** 2025-07-04

**Authors:** Menthy Denayer, Eligia Alfio, María Alejandra Díaz, Massimo Sartori, Friedl De Groote, Kevin De Pauw, Tom Verstraten

**Affiliations:** 1 Robotics & Multibody Mechanics (R&MM) Research Group, Vrije Universiteit Brussel, Pleinlaan 2, Elsene, 1050 Belgium; 2 Flanders Make, Brussels, Belgium; 3 Human Physiology and Sports Physiotherapy Research Group, Vrije Universiteit Brussel, Pleinlaan 2, Elsene, 1050 Belgium; 4 Department of Biomechanical Engineering, University of Twente, Enschede, 7522 NB Netherlands; 5 Department of Movement Sciences, KU Leuven, Leuven, 3001 Belgium

**Keywords:** Gait, Human, Locomotion, Musculoskeletal simulations, Optimization, Predictive, Reinforcement learning, Central pattern generators, Muscle-reflexes

## Abstract

This PRISMA systematic review covers the literature on predictive, musculoskeletal simulations. First, we define predictive movement for musculoskeletal systems, as the current literature suffers from inconsistent nomenclature. We distinguish two methods of prediction. The first uses neural models, like muscle-reflex-based and central pattern generator models. The second uses optimization, to make up for the lack of a neural model, like optimal control and deep reinforcement learning. For each method, we illustrate the main concepts and report accuracies, simulation times and limitations. Moreover, we identified key works over the past 50 years, which are fundamental for the current state-of-the-art. The majority of works employ optimization. We recognize six classes of cost function terms and note they are often combined using linear combinations. We describe musculoskeletal models, their muscle model, ground contact model and personalization. Similarly, we identify key software like OpenSim and SCONE. Additionally, we provide an overview of simulated movements, pathologies and assistive devices. We emphasize the difference in tracking simulations and prediction, while clarifying the benefits of using experimental data to predict movement. Finally, we call for quantitative validation to establish comprehensive comparisons between methods. To this end, we share a list of works open-sourcing their codes.

**Supplementary Information:**

The online version contains supplementary material available at 10.1186/s12984-025-01686-w.

## Background

Musculoskeletal simulations use virtual models of the human body to study different types of motion, like walking [1], jumping [2], reaching [3] and more. Typically, musculoskeletal (MSK) models are driven by experimental data, like body kinematics [4, 5], ground reaction forces or electromyography (EMG) signals [6]. However, predictive simulations, that can extend beyond available experimental data, are required to predict the effects of assistive devices, surgeries and pathologies, and to understand the human neural controller [7]. A clear definition of simulations, predicting kinematics and kinetics of the MSK system, is still missing in the literature.

Since 1970, researchers have developed different approaches to predictive simulations, starting with Hatze et al. [8] using optimization to move a model of the right leg from an initial to a final point in the shortest time possible. The first objective of this review (RO1) is to overview existing methods to predict the movement and kinetics of MSK systems. Secondly (RO2), we create a timeline of key historical developments.

Different software platforms support MSK simulations, including OpenSim built on Simbody [9], SCONE [10], AnyBody [11] and physics-based engines like MuJoCo [12], with projects such as MyoSuite [13]. The MSK models themselves also vary in personalization, number of degrees of freedom, number of muscles and contact models. We discuss the existing MSK software and modelling options (RO3).

To assess their ability to make accurate predictions, the simulations need to be validated against experimental data, which includes kinematics data from motion capture systems, ground reaction forces recorded with force plates or EMG data. We explore validation methods (RO4) and discuss common limitations and errors compared to measured data (RO5).

These simulations serve various applications, from designing assistive devices [14, 15], to understanding pathologies [16, 17], predicting surgical outcomes [18] and stuyding performance in sports [19, 20]. We survey current applications with predictive MSK simulations (RO6).

The present review focuses on the prediction of movement (kinematics, kinetics) of multibody, MSK models. Simulations predicting physiological variables, like muscle forces, while tracking experimental data are discarded. Additionally, we focus on MSK models, where muscles form the main actuators. The review addresses three gaps in the existing literature: (1) previous reviews often focus on one subdomain of solutions, like optimal control [21, 22] or reinforcement learning [23, 24], with De Groote and Falisse [25] discussing both optimal control and reinforcement learning, while we provide a comprehensive overview, including model-based methods; (2) we address validation aspects lacking in previous works [26]; and (3) we follow PRISMA guidelines, unlike many existing narrative reviews [25, 27].

We aim to answer the following four research questions.

- *Q1: How can we define movement prediction of a multibody, musculoskeletal system?* We want to define the prediction of kinematics and kinetics of a MSK system and how we can distinguish between solutions that are predicting physiological variables like muscle forces, based solely on experimental data.
- *Q2: What are the limitations of current MSK models?* We give an overview of the used MSK models, their degrees of freedom, muscles and ground contact models, and discuss their limits.
- *Q3: How can we generate predictive motion?* We make a distinction between neuromusculoskeletal (NMSK) models and optimization-based approaches, and discuss their scope and application potential.
- *Q4: What are current validation practices?* We mention validation data, metrics and recommendations. Additionally, we compare current validation practices to the ones proposed by Lund et al. [28] for MSK simulations.

The review paper is organized in seven sections: “Methodology”, predictive methods “Techniques for predictive simulations”, MSK models “Musculoskeletal models”, validation “Accuracy & validation”, applications “Applications & development” and “Discussion”. Supplementary material is available online.

## Methodology

**Fig. 1:**
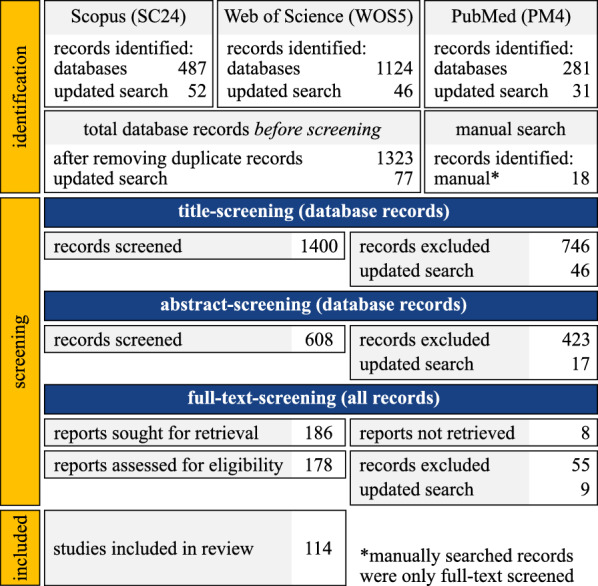
PRISMA flowchart illustrating the review process. Overall, we included 114 papers in the review. We updated the search results once with papers from 2024, with a final date of 23/12/2024

The review was conducted according to PRISMA guidelines [29]. Figure 1 gives an overview of the process. The research questions and objectives were defined according to the Population, Concept and Context (PCC) approach. We replaced population by problem, as often done for the Population, Intervention, Control and Outcome (PICO) method [30].

### Search strategy

We derived the search query in Table 1 to identify all papers describing predictive musculoskeletal simulations, for no specific movement type. We used an iterative process, adding or removing keywords, until most of the identified papers were relevant to the review’s topic. We also verified that important milestones (Fig. 3) were included inside the identified papers.

**Table 1:** Search query used in Scopus

musculoskel* OR musculo-skel* OR neuromech* OR neuromusc*	AND
simulat* OR “muscle?reflex model*” OR framework	AND
predict* OR mimic* OR synthesi* OR imitat* OR replicat* OR reproduc* OR produce OR learn* OR generat*	W/10
motion* OR move* OR locomotion OR gait OR ascen* OR descen* OR "motor skill*" OR kinematic*	AND
optim* OR minim* OR “stab*” OR steady OR “symmetr*” OR healthy OR impaired OR pathologic* OR assisted	AND
train* OR control OR feedback* OR reinforcement OR forward OR dynamic	AND
human* OR person* OR subject* OR biped*	AND NOT
insect* OR fluid* OR “lung*” OR “eye*” OR “functional electrical stimulation”	AND
PUBYEAR < 23/12/2024	AND
LIMIT-TO (DOCTYPE , “ar”) OR LIMIT-TO (DOCTYPE , “cp”)	AND
LIMIT-TO (LANGUAGE , “English”)	

For the Web of Science and PubMed databases, “W/10” was replaced by “AND”

We searched three databases: Scopus, Web of Science and PubMed. In Scopus, we required keywords related to prediction to be within 10 words of terms describing motion or the predicted variable. As this operator is only available in Scopus, we replaced it by “and” for the other two databases. We looked for papers published before the year 2024, without a lower threshold. We repeated the process once for papers published in 2024, with a final date of 23/12/2024 (Fig. 1). The papers should be articles (“ar”) or conference papers (“cp”) and be written in the English language. We imported the papers into Zotero and removed the duplicates.

The review process consisted of three steps. The exclusion criteria are mentioned in “Criteria” section. Firstly, all 1323 identified articles were title-screened. As a result, 746 papers were excluded. Detailed information about the excluded papers can be found in the supplementary material.

The remaining 746 papers were abstract-screened by two independent reviewers, in two rounds. First, both reviewers screened all abstracts, including the paper or assigning an exclusion reason. During the first round, the Cohen’s Kappa ($\kappa$) [31] and Intra Class Correlation coefficients (*r*) [32] were 0.50 and 0.48 respectively. The values show moderate agreement [31] and are further discussed in the “Discussion” section. Formulas for both metrics are added in the supplementary material. After both reviewers went through the conflicting papers again, the metrics increased to a value of $\kappa =0.91$ and $r = 0.88$, showing almost perfect agreement [31]. We excluded 423 papers after the abstract screening (supplementary material).

From the remaining 186 papers (168 from the abstract-title screening and 18 from a manual search), 8 works were not found, and 178 were full-text-screened. The manual search contained 10 papers, which were identified before the review process, but were not found during the identification. Additionally, 8 papers were added from the SCONE publications database [33], as this is a popular software for predictive simulations. Two independent reviewers read each paper and collected the main data entries (“Data collection & organization” section). We excluded 64 papers, based on the criteria defined in the “Criteria” section: $25\%$ dealt with out-of-scope motions, $30\%$ was not predictive, $19\%$ used a torque-driven model, $19\%$ did not focus on presenting or using predictive simulations, $5\%$ were reviews and $3\%$ were duplicates. In total, we included 114 papers inside the review. The inter-reader reliability metrics for the full-text screening show substantial agreement ($\kappa = 0.76$, $r = 0.88$) [31].

### Criteria

An extended list of criteria can be found in the supplementary material.

We focus on lower limb motion. Cyclic motions, like cycling [34] were excluded, as the bike’s motion largely defines the body kinematics. We also excluded studies tracking data of highly specific motions (e.g. skiing [35]). Finally, we excluded static balance control and posture control movements. These have already been covered extensively by another review [26].

The papers should use MSK models, actuated by muscles. Torque-driven models [36], used in robotic studies or avatars without muscles are excluded.

The motion should be predictive. Thus, papers using pre-defined excitation signals or joint angles [37], or tracking experimental data [38] are excluded. However, some papers ($\pm 18\%$) use experimental data to find an initial estimate of muscle excitations [39] or to tune controller parameters [40] and are included, as long as the tracking terms inside the cost function do not dominate the remaining terms [41].

Finally, the included papers need to introduce a method for predictive motion or use it to study the effect of, for example, pathologies or assistive devices. Papers introducing software without a focus on generating predictive motion are excluded. Similarly, papers using predictive motion without mentioning the effect on kinematics (joint angles) are also excluded.

### Data collection & organization

We collected data related to the method of achieving predictive motion, the software and implementation, the simulated movement and the MSK model. A table containing the collected data can be found inside the supplementary material.

Papers were classified based on their implementation of the neural controller of the MSK system. About half ($58\%$) created a NMSK model, extending the MSK model with a policy, defining the muscles’ excitation based on some inputs. The policy can be a physiological model (model-based) or a neural network (black box). In the former case, studies use muscle-reflex models ($32\%$ of included papers), mostly based on the work by Geyer and Herr [42], and central pattern generators (CPGs) ($16\%$). The neural model parameters are found through manual tuning or optimization. Alternatively, $29\%$ of papers use deep reinforcement learning (DRL) to find the optimal neural network weights. The other $42\%$ of works use optimal control (OC) to find the unknown muscle excitations without a neural controller, which is currently the most common method of motion prediction (Fig. 3).

**Fig. 2:**
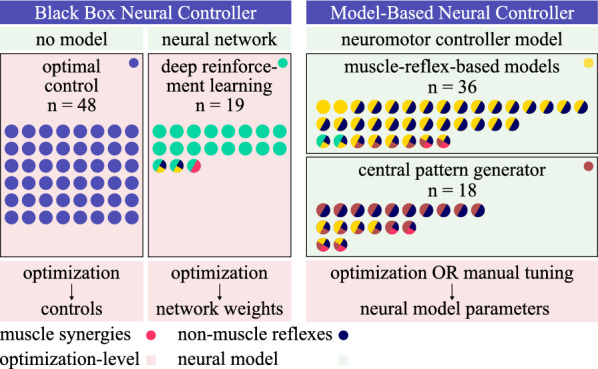
Overview of the methods to generate predictive motion. Each dot represents an included paper. Multi-coloured dots combine multiple methods. Green boxes contain neural models, while red boxes contain optimization solutions. The neural control models often combine several concepts

We distinguish works using a mechanistic model of the neural controller (muscle-reflexes and CPGs), from those using a black box controller and optimization (OC and DRL) to make up for the lack of such a model (Fig. 2).

Additionally some papers ($4\%$) use muscle synergies or include non-muscle reflexes (e.g. trunk tilt) inside their model ($40\%$). One paper employs a hybrid zero dynamics model (not represented in Fig. 2) [43], and two papers combine both approaches (a muscle-reflex model and DRL) [44, 45].

## Techniques for predictive simulations

### Black Box neural controller

#### Optimal control

Optimal control formulates the problem of motion prediction as an optimization problem, solving for the unknown time trajectories of muscle excitations. The system is defined by the MSK model and described by its states *x*(*t*) (i.e. joint angles, velocities) and its control inputs *u*(*t*) (muscle excitations) [46]:

1 $$\begin{aligned} \begin{array}{ll} \min \limits _{x(t), u(t)}& \mathcal {O}(x(t),u(t)) \\ \mathrm {s.t.} & \dot{x}(t) = f(x(t),u(t)) \\ & 0 \le u(t) \le 1 \\ & \mathcal {T}_i(x(t),u(t)) = 0, \end{array} \end{aligned}$$

where $\mathcal {O}$ is the objective function, to be minimized, *f* contains the system dynamics, and $\mathcal {T}_i$ are the task constraints, like periodicity, initial conditions or speed constraints [46].

**Fig. 3:**
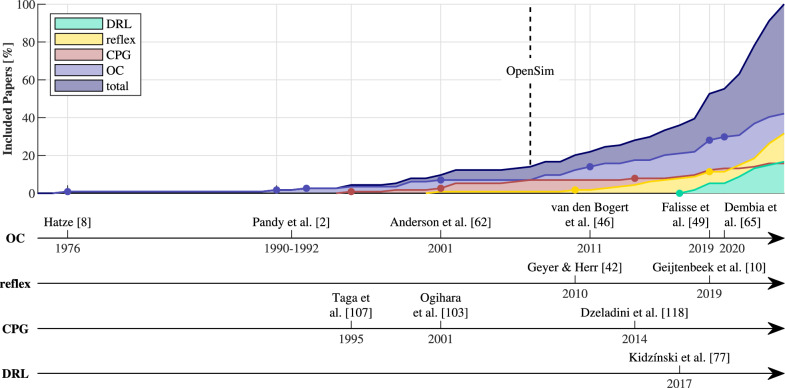
Top: cumulative percentage of included papers for each publication year and solution type (DRL: Deep Reinforcement Learning, reflex: muscle-reflex-based models, CPG: Central Pattern Generators and OC: Optimal Control). Coloured dots represent milestones. The release of OpenSim (dotted line) led to an increase in studies. Bottom: timeline of important works

Hatze et al. [8] pioneered this approach in 1976 (Fig. 3), optimizing muscle excitation times for a 2 DOF model of the right leg with 5 muscles, to achieve the bending movement of the leg in the shortest time possible. They used differential dynamic programming, an indirect method, to solve a boundary-value problem. However, these methods do not scale well for more complex (3D) models [46]. Hence, direct methods, like direct multiple shooting [47, 48] and direct collocation [39, 46] emerged. Direct methods parametrize the control and/or state, transforming the OC problem into a nonlinear programming (NLP) problem [21], solvable using gradient-based algorithms [49], or heuristic methods like simulated annealing [50, 51] and genetic algorithms [50, 52–57]. Popular NLP solvers include Sparse Nonlinear Optimizer [58] and Interior-Point Optimizer [59].

Next, Pandy et al. [2, 60] applied OC to jumping movements and used parameter optimization in 1992 to transform the OC problem into an nonlinear programming (NLP) problem [61], similar to the direct methods mentioned above. In 2001, Anderson et al. [62] extended this approach to walking, which is the main studied movement in predictive simulations (“Applications & development” section). van den Bogert et al. [46] implemented direct collocation (DC) with OpenSim models, which Porsa et al. [63] demonstrated to be up to 249 times faster than direct shooting methods, achieving the same solution. Direct collocation considers the discretized system dynamics implicitly as a constraint, allowing for higher numerical efficiency. Before, shooting methods were often used, which use time marching to enforce the system dynamics. Direct collocation improves numerical efficiency for stiff differential equations, which would require small integration time steps in the case of direct shooting. Finally, Falisse et al. [49] further improved efficiency by combining direct collocation with algorithmic differentiation (AD) through an interface between CasADi [64], a tool for formulating NLP problems and algorithmic differentiation, and OpenSim. This led to specialized software developments like OpenSim MoCo (2020, OpenSim and DC) [65] and PredSim (2024, OpenSim, DC and AD) [66].

Aside from the time histories of muscle excitations, other optimization variables include initial states (e.g. joint velocities) [51], the total simulation time [19, 67] or control signals and parameters of an added assistive device [68, 69]. Li et al. [43] use hybrid zero dynamics to generate a trajectory for a transfemoral prosthesis, based on a MSK model. They use direct collocation to find the optimal set of Bézier coefficients, defining the desired system outputs, i.e. joint angles and the forward velocity, minimizing the cost of transport (COT). Using a feedback linearizing controller, the error between the system outputs and the desired values is driven to zero.

Additionally, some papers use bilevel optimization or inverse optimal control to optimize the cost function weights inside a second optimization loop [54, 55, 70]. Nguyen et al. [54, 55] use two separate cost functions. A lower-level optimization solves for the predictive walking motion. The upper-level optimization finds the optimal weights, such that the lower-level predictions best replicates the experimental data. They use direct collocation to solve the lower-level problem and a genetic algorithm to find the optimal weights. Tomasi et al. [70] introduced a weighted Chebyshev norm $\mathcal {O}_C$ to aggregate the objective terms.

2 $$\begin{aligned} \min _{x(t),u(t)} \mathcal {O}_C(x(t),u(t),w) = \max _{i=1,...,m} w_i \tilde{\mathcal {O}}_i(x(t),u(t)), \end{aligned}$$

where the objective terms $\tilde{\mathcal {O}}_i$ are scaled to vary between 0 and 1 along the Pareto optimal front and $\sum w_i = 1$. The objective function can take on values along the Pareto optimal front and the weights are a better representation of the relative importance of each term [70]. Finally, Weng et al. [71] developed Adaptive Reference Optimal Control, using a gradient-based optimization for tuning the weights.

In 2001, Anderson et al. [62] required 10,000 h CPU time to simulate a 3D model of 54 muscles. Ackermann et al. [72] simulated a 2D, 16 muscle, MSK model in 35 min, in 2010. van den Bogert et al. [46, 73] and Lee et al. [74] report simulation times as low as 5–10 min or 15 s, with 16 and 9 muscles respectively, for a 2D MSK model. Recently, several papers, simulating 2D MSK models with 12–18 muscles, reduced the optimization time below 1 h [20, 47, 49, 72]. In 2014, Lee et al. [75] simulated a 3D model with 120 muscles in 6–9 h. They formulated the muscle redundancy problem as a quadratic program, solved using Quadprog++ [76], by linearizing the muscle dynamics equations. Additionally, they used CMA-ES to modulate the reference motion (trajectory optimization) to adapt to new conditions. Recent papers, simulating more than 90 muscles [67, 70] and using DC, achieve simulation times between 1-9 h, with Falisse et al. [39] even simulating a 3D model with 92 muscles in 36 min. They use DC, implicit differential equations, and AD to achieve efficient simulations. They found that using AD can speed up the simulation time by a factor of 60, compared to using finite differences. However, this time gain also depends on the cost function and the number of dependent variables [49].

#### (Deep) reinforcement learning

DRL was first applied to MSK models in 2017, through the “NEURIPS: learning to run challenge” by Kidzínski et al. [77] (Fig. 3). Before, DRL had only been applied to basic, torque-driven models, without biologically accurate actuators.

In DRL, the agent’s behaviour follows a Markov Decision Process. The agent (MSK model) interacts with the environment (physics simulator) through actions, based on observations. The action is sampled from a learned policy $\pi (a_t | s_t)$, a function of the state $s_t$. Each action $a_t$ leads to a new state $s_{t+1}$, according to the system’s dynamics $\mathcal {P}(s_{t+1}|s_t, a_t)$, earning a reward $r_t = \mathcal {R}(s_t, a_t)$ [78]. The agent maximizes their reward, by optimizing the policy [79].

The policy is a user-defined function. In most papers, this is a deep neural network. However, it can also be a model-based controller (“Model-based neural controller” section) [45]. The network consists of different layers, each containing neurons. The state of the MSK system, including the information from the environment (ground reaction forces) and muscle states (activation, lengths, force, velocities) is the input to this network [80]. Each neuron $v_i$ uses the signals from the neurons of the previous layer $x_i$ to compute an output $y_i(v_i) = f(x_i w_i)$, where *f* is called the activation function. The hyperbolic tangent [81] or ReLU [82] functions are often used to transform the inputs into a value between 0 and 1, or a strictly positive value. $x_i$ and $w_i$ denote the inputs and weights between the current and previous neurons, respectively. The weights $w_i$ of the network are determined through a RL algorithm.

The standard policy gradient algorithm is simple but suffers from instability and inefficiency [83]. The Proximal Policy Optimization and Trust Region Policy Optimization algorithms address the stability issue by restricting the changes in the policy after each iteration, and are used in about $63\%$ of DRL papers. The remaining papers ($37\%$) use an off-policy algorithm, like Deep Deterministic Policy Gradient or Soft Actor-Critic to improve the sample efficiency. These algorithms estimate the policy gradient by fitting a Q-function, representing the expected reward after taking an action [83].

Kidzinski et al. [77] released the “OpenSim-RL” library in 2017, which is used by several papers [45, 77–80, 83–86]. It combines the OpenAI Gym environment with OpenSim. However, it requires more computational resources compared to existing Gym environments in MuJoCo [77].

Some papers use curriculum learning (CL) to train the DRL agent [86, 87]. CL gradually encodes the progress of motor skill development by dividing the training process into different phases, becoming increasingly more difficult. This allows the agent to acquire complex motor skills more efficiently [86]. For example, Weng et al. [86] first teach their agent to stand, next to walk, move forward and eventually tune their gait. For each training phase, they enable different terms of the reward function to encourage different types of behaviour. In the first phase, the agent aims to survive and prevent hyperextension. After, terms encouraging moving forward, stability or minimizing effort are included. Qin et al. [87] use CL to escape local minimum solutions. They implement a control target signal to make the agent move to a desired goal, within a certain (distance & orientation) range. During the CL training phases, they increase the angle range, to improve the agent’s ability to distinguish orientation and turn towards the target. As a result, the agent can reach the target in $96\%$ of cases with CL, for a turn of $50^{\circ }$, while the agent succeeds in only $36\%$ of cases without CL.

While most papers output the muscle excitations as the output of the neural network [81, 82, 86], other employ a hierarchical framework, with multiple policies trained simultaneously [14, 18, 87, 88]. The trajectory generation network outputs the target joint angles, which are transformed by a proportional-derivative controller into joint torques or accelerations, for the muscle coordination network, which outputs the required muscle excitations. Additionally, Luo et al. [14] train yet another network to determine the required joint torques for a lower-limb exoskeleton in parallel. Jiang et al. [89] train two neural networks to estimate realistic joint torque limits and the metabolic cost, based on joint-space information. They use their learned networks to train a torque-based model to walk while minimizing its COT. Their method of converting muscle-based actuation models into physiologically plausible joint-space problems can also be used in OC solutions.

In terms of the simulation time, existing solutions vary between a couple of hours [78, 86, 89] and days [81, 85, 87, 88]. Weng et al. [86] manage to train a DRL 2D model with 18 muscles in 1.5 h, using PPO and CL, achieving a mean correlation coefficient above 0.92 for the hip, knee and ankle. However, the trained model still displays toe-walking and not enough knee flexion before foot strike. Lee et al. [18] and Park et al. [88] train a model using PPO with 346 and 304 muscles, requiring 4–5 days and 12–36 h of training time, respectively. While Lee et al. [18] use imitation learning to achieve a more realistic gait pattern, Park et al. [88] developed a tool to generate different (pathological) gait patterns, without the use of experimental data.

### Model-based neural controller

#### Muscle-reflex-based methods

Geyer and Herr [42] introduced muscle-reflex-controlled models in 2010 (Fig. 3). They divide the gait into two parts: stance and swing. During both cycles, the leg muscles are controlled, including the soleus muscle (SOL), vasti muscle group (VAS), gastrocnemius muscle (GAS), tibialis anterior muscle (TA), hip flexor muscles (HFL), gluteus muscle (GLU) and hamstring muscles (HAM). Each muscle is activated based on local feedback, like the muscle’s length and force. For the leg muscles, muscle *m*’s stimulation is a combination of force ($S_m^F(t)$) and length feedback ($S_m^L(t)$):

3 $$\begin{aligned} S_m^F(t)&= S_{0,m} \pm G_{m}^F F_m (t - \Delta t_{m}), \end{aligned}$$

4 $$\begin{aligned} S_m^L(t)&= S_{0,m} \pm G_{m}^L (l_{CE,m} - l_{off,m}) (t - \Delta t_{m}), \end{aligned}$$

where $S_{0,m}$ is a constant prestimulation, $G_{m}$ represents a gain, $F_m$ is the muscle force, $\Delta t_{m}$ denotes a time-delay, $l_{CE,m}$ is the muscle’s fiber length and $l_{off,m}$ is a length offset. The force and length feedback can be positive or negative, depending on the muscle’s function. Additionally, one muscle’s stimulation can include feedback terms from other muscles. For example, the TA contains a negative force-feedback term from the soleus, to avoid the muscles fighting when flexing the foot. Finally, some muscles, like VAS contain an inhibition term $S_m^I(t)$, to prevent overextension.

5 $$\begin{aligned} S_{m}^I(t) = - k_{\varphi } \Delta \varphi _k (t - \Delta t_k), \end{aligned}$$

where $k_{\varphi }$ is a proportional gain and $\Delta \varphi _k$ denotes the distance of the joint angle $\varphi _k$ to its limit. For example, in the case of the VAS muscle, $\Delta \varphi _k = \varphi _k - 170^{\circ }$, to prevent knee hyperextension. This term is only active when $\Delta \varphi _k> 0$. The hip muscles (GLU, HFL) use a proportional-derivative signal of the trunk’s forward lean angle for balance. Additionally, the stimulation is modulated by the amount of weight the leg bears, since the hip torques can only balance the trunk if the leg bears sufficient weight on the ground. Finally, to initiate swing, the VAS muscle’s positive force feedback is inhibited by adding a term, proportional to the weight the contralateral leg bears: $-k_{bw} |F_{leg}^{contra}|$.

Geyer and Herr [42] tuned the gains and time-delay parameters manually to match their mechanical requirements. However, recent works [15, 90] use optimization algorithms like Covariance Matrix Adaptation-Evolutionary Algorithm (CMA-ES) to solve for the neural model’s parameters that optimize an objective function. Antonova et al. [91] use Bayesian optimization, to improve sample efficiency. They score the optimized gait using gait determinants, dealing with the conservation of energy and maintaining forward momentum. The total score is used inside a squared exponential kernel to assess the similarity between solutions for different sets of parameters. In 2019, Geijtenbeek launched SCONE [10], enabling users to explore predictive simulations with different control strategies, including the Geyer and Herr muscle-reflex-based controller, combined with optimization, like CMA-ES.

The neural parameters are correlated to certain gait characteristics. Russo et al. [92] used a controller based on Ong et al. [93], and found 9 reflex parameters to modulate the human gait (i.e. changing its speed, step length and step duration). The positive force feedback gain of the GAS and SOL, for example, is highly correlated to the step length ($R>0.70$) and speed ($R>0.89$), as these muscles give the main gait propulsion, during stance.

Several papers have built upon the work of Geyer and Herr. Etoh et al. [94] increased the number of muscles to 70 and used a feedforward and feedback control with delay elements to account for muscle tone. Song et al. [7, 95] extended the model to 3D, increased the number of muscles, organized the neural control circuitry into 10 spinal reflex modules and added a higher control layer adjusting the foot placement. Ramadan et al. [96] extend the muscle-based reflex module with a supraspinal layer to add goal-directed movements. Geijtenbeek et al. [40] consider four phases: stance, lift-off, swing and stance preparation, instead of two, similar to Wang et al. [97]. The former also use muscle-based feature control instead of length-based feedback, to find the muscle excitations driving the segments to their desired locations. Three papers even use five different high-level states, three for stance and two for swing [98, 99], with Ong et al. [93] building on the works of Geyer and Herr [42], Song and Geyer [7] and Wang et al. [97], and also including muscle-velocity feedback.

Muscle-reflex models are also used to predict movements, different from walking. Van Der Kruk and Geijtenbeek [100, 101] used a muscle-reflex controller to predict sit-to-walk movements. They combined a two state stand-up controller, for standing, with an extended version of the Geyer and Herr gait controller for walking.

The optimization process of the reflex parameters mainly determines the simulation time. Computation times range from 2–20 h [40, 91, 97, 98, 102, 103] to multiple days [7, 94, 104, 105].

#### Central pattern generators

CPGs are believed to generate neural stimuli at the lower levels of the central nervous system (i.e. the spinal cord) [106]. Taga et al. [107] laid an important foundation for using CPGs with MSK models. They mention that basic gait emerges from the entrainment between the rhythmic activity of the neural system and the rhythmic movement of the MSK agent. CPGs can generate signals autonomously, without sensory feedback signals or inputs from a higher-level controller (i.e. the brain) [102]. Although several implementations are used in the literature to represent the CPGs, a rhythm generator (RG) is typically the main component. The RG consists of a network of neural oscillators generating a rhythmic signal, governed by a set of differential equations [107]:

6 $$\begin{aligned} \dot{x} = \varphi (x,u_{SN},u_{MLR}), \end{aligned}$$

where *x* represents the state of the neural oscillator, $u_{SN}$ a feedback signal from the proprioceptive or exteroceptive sensors and $u_{MLR}$ a signal from the higher-level controller. The sensory feedback $u_{SN}$ and higher-level control signals $u_{MLR}$ are entirely optional [102]. The CPG can create feedforward signals autonomously. The shape of the differential equation $\varphi$ is different between studies. The Matsuoka model is often used [103, 108]. Similarly, the number of neural oscillators and CPG modules differs between works: Taga et al. [106, 107] use one neural oscillator for each muscle, other works use 2 coupled, neural oscillators for each degree of freedom [109–111], or similarly, use half-center CPG modules, consisting of 2 coupled oscillators, stimulating the flexor and extensor muscles [102, 112–114].

The output of the RG can be used directly to stimulate the muscles or compute the muscle torques [106]. Other works include a pattern formation (PF) network. Different implementations exist for the PF layer. The PF layers have three functions [102]: (1) to integrate exteroceptive signals (e.g. from the body angles or ground pressure); (2) to react to signals from a higher-level controller; and (3) to transform the stimulus signal to a range between zero and one. Feedback signals can be integrated into the RG, to alter the locomotor rhythm (e.g. phase resetting or shifting) or at the PF level, changing the activation amplitude and timing of phase transitions [115]. So, while the RG dictates the overall pace and phase of the stimulation, the PF layer decides on the stimulation pattern and shape [113, 116].

As mentioned, proprioceptive and exteroceptive signals can also be incorporated into the CPG module. The proprioceptive feedback includes spinal reflexes like Ia, Ib and II afferents, modelled by muscle velocity, length and force feedback respectively [103, 116]. For example, Ogihara and Yamazaki [103] introduce muscle feedback into the CPG module in 2001. The feedback includes the muscle state, body angles, joint angles and the detection of plantar pressure [116]. It can be incorporated into the RG module [103, 106, 107, 111, 117], the PF module [102, 112, 113] or a separate motor neuron (MN) layer [17, 108, 114]. Additionally, Di Russo et al. [116] also include a PD controller for balance. The works using sensory feedback information are indicated as using reflexes in Fig. 2. Finally, the higher-level controller (i.e. the brain) can also send signals to the CPG module. These signals are used to change the locomotor pattern, depending on the task requirement, or locomotion speed [106, 107, 112], and they vary the frequency of the generated patterns, perform phase deletion or clamp the control signals [113].

Finally, $56\%$ of the papers using CPG models, use optimization to tune the model’s parameters. This is done with genetic algorithms [17, 103, 108, 110, 111], which are a subset of evolutionary algorithms, based on biological principles, like mutation, crossover and selection. Others use particle swarm optimization (PSO) [118] and CMA-ES [116]. Similar to the muscle-reflex-based models, the major simulation time is related to the optimization process, which can take between 12–24 h [109, 111].

#### Combined neural models

Some papers combine muscle-reflexes with CPGs, implementing spinal reflexes, represented by the muscle-length, velocity and force feedback and combining them with a CPG module [112, 116].

Dzeladini et al. [118] and Haeufle et al. [119] specifically shape the CPG signal to match the predicted muscle excitations by the Geyer and Herr [42] model, instead of using a RG. They apply the feedback signals from steady-state walking, as a repeating feedforward signal [119]. After, the feedforward CPG signal is linearly combined with the muscle-reflex feedback signal [118]:

7 $$\begin{aligned} u_m(t) = u_m^{FF}(t) + w_m( u_m^{FB} - u_m^{FF}), \end{aligned}$$

where $u_m(t)$ represents the muscle excitation, $u_m^{FF}$ the pre-recorded, feedforward signal, $u_m^{FB}$ the feedback signal based on the Geyer and Herr model and $w_m$ a weight. The feedforward signal is synchronized with the gait at touchdown or takeoff [118]. The feedforward signals increase the stimulation of antigravity muscles (VAL, SOL, GAS) in anticipation of the drop in the ground level [119] .

### Muscle synergies

Five studies use the muscle synergy hypothesis in their controller (Fig. 2), taking advantage of the idea that a linear combination of a limited number of basic signals can produce a large portion of motor commands for the muscles [120]. In human walking, five primitive synergy patterns are already enough to generate a large portion of the muscle excitations [116, 120, 121]:

8 $$\begin{aligned} u_{syn} = \sum _{i=1}^5 w_{m,i} \Lambda _i p_i(\phi ), \end{aligned}$$

where $w_{m,i}$ are weights given to pattern $p_i$ and motor neuron *m*, $\Lambda _i$ are tuning parameters for the pattern’s amplitude to generate different speeds and gaits [121] and $p_i(\phi )$ is the primitive pattern, as a function of the gait phase $\phi$. The latter is determined through a set of differential equations, as described above. The weights can be determined through optimization [116]. The patterns can be rectangular pulses [120, 121] or a bell-shaped waveform, implemented as a raised-cosine function [116]. Shachykov et al. [112] use muscle synergies by creating only 2 CPG modules per leg, to control the 80 modelled muscles. They split the muscles into four groups of two non-spanned pairs. Each CPG controls simultaneously activated muscle groups.

Finally, Zuo et al. [122] use muscle synergies and DRL, training a policy and an encoder-decoder structure, to transform a low dimensional muscle cluster action into the activation of all individual muscles.

### Combined approaches

Wang et al. [44] combine muscle-reflex feedback with DRL. The muscle-reflexes with optimized gain parameters achieve stable walking. Additionally, the human model is trained using DRL to detect changes in the environment and adopt its controller accordingly. The policy, a neural network, adopts the muscle-reflex parameters, like the trunk balance feedback parameters, to account for the changing terrain. The DRL policy allows the model to walk for 435 m on mixed terrain (flat, slope and wavy), while the Geyer and Herr controller is only able to walk for 239 m, when optimized with mixed data. Similarly, Su et al. [45] use a muscle-reflex model, based on Song and Geyer [7], as the policy and RL to tune the gain, time delay and trunk reference parameters.

### Optimization goals

Only 10 papers do not use any optimization [42, 106, 107, 113, 114, 117, 119–121, 123]. Optimization is used for three main reasons. Firstly, in works using OC, to find a time series of muscle excitations, optimizing a certain objective function. Secondly, to train a model using reinforcement learning (RL) and tune the neural network weights. Finally, $32\%$ of included papers use optimization to tune the parameters of their neural model.

#### Objective functions are mostly linear combinations

**Fig. 4:**
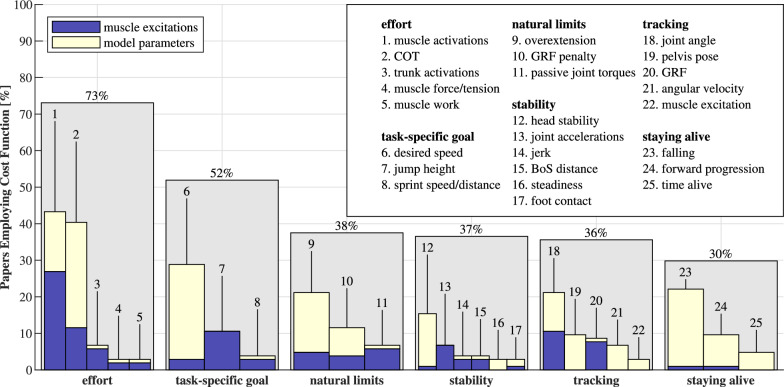
Cost function terms organized according to their occurrence for the papers using cost functions: effort terms reducing the model’s energy, task-specific goals promoting specific objectives, natural limits penalizing unrealistic physiological values, stability promoting steadiness, tracking terms following (experimental) data and terms encouraging the model to keep moving. The terms are further divided depending on the optimization variables (muscle excitations or neural model parameters)

A major part of the optimization process consists of defining the objective function $\mathcal {O}$—or cost function whenever the function is minimized. This is typically (in $90\%$ of papers using a cost function) a linear combination of scalar objective terms:

9 $$\begin{aligned} \mathcal {O} = \sum _{i = 1}^N w_i \mathcal {O}_i = w_1 \mathcal {O}_1 + w_2 \mathcal {O}_2 + ... + w_N \mathcal {O}_N, \end{aligned}$$

where $\mathcal {O}_i$ represents the $i{\text{th}}$ objective term and the coefficients $w_i$ are called the weights. The latter determine the relative importance of the individual objectives. The weights can also be negative, resulting in a penalty term [110]. They can either be chosen manually or optimized as part of a second optimization [54, 55, 70, 71]. Alternative combinations of individual objectives are possible [18, 87, 88]. Tomasi et al. [70] mention the pitfalls and considerations, when combining the objectives linearly into a scalar value, for OC. In DRL the objective terms (Fig. 4) are often represented using exponentials $\mathcal {O} = \sum _i w_i e^{\alpha _i \mathcal {O}_i}$. When the agent deviates from the desired behaviour, it is penalized exponentially. Works using tracking terms inside the objective may favour using exponential tracking terms [78].

The most common objective terms are: effort, promoting task-specific goals, tracking experimental data, preventing unrealistically high physiological values, rewarding stability and encouraging the model to keep moving (Fig. 4).

#### Effort is the most common objective

Papers typically assume that humans perform their movement to minimize their energy consumption, as is the consensus for well-practised motor tasks [24]. Hence, the primary goal of most objective functions ($73\%$ of papers using optimization) is to reduce the model’s effort or energy. Two terms commonly represent the model’s energy. Muscle activations, put to a certain power, summed over all modelled muscles is the most prominent ($43\%$) representation of effort.

10 $$\begin{aligned} \textrm{effort} \approx \sum _i (a_i(t))^p \end{aligned}$$

The activations can be squared ($p=2$), cubed ($p=3$), or even higher powers, like $p=10$ [16]. Ackermann and van den Bogert [72] compared the effect of different exponents *p*. They distinguish between cost functions with higher *p*-values ($2 \le p$) without muscle volume scaling and lower *p*-values ($p \le 3$) with muscle volume scaling. The former objectives represent simulations minimizing the model’s fatigue, penalizing primarily the highest muscle activations. The latter objectives correspond to simulations reducing the model’s effort, which is related to the activated muscle volume. The largest difference in terms of kinematics lies in the reduced knee flexion angle, during initial and mid-stance, for the effort-like simulations. These lead to a more straight-leg gait pattern and higher impact forces as a result.

Alternatively, muscle work [56, 90] or muscle forces [60, 75] are also used as an effort metric. Additionally, activations of the trunk and/or arms actuators are also minimized, whenever the model includes a flexible trunk and/or arms [16, 39, 67, 70, 71, 97, 124].

**Fig. 5:**
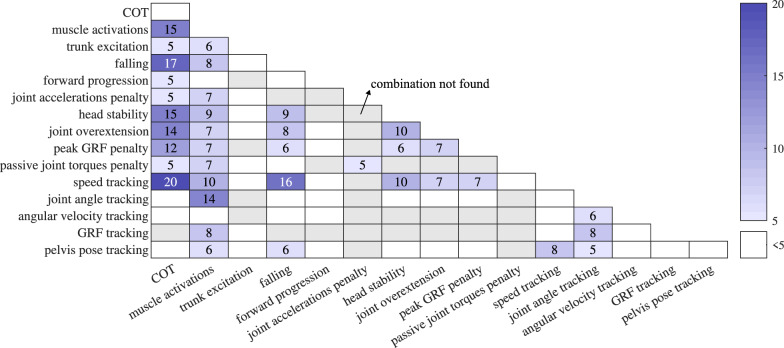
Number of papers combining cost function terms. Grey tiles represent cases that did not occur inside the screened papers. Only pairs with more than 4 occurrences are shown. The effort terms (COT, metabolic rate and muscle activations) are often combined. COT is also often combined with speed tracking terms and terms preventing falling. Joint angle tracking typically occurs in papers minimizing the muscle activations, and is often combined with GRF tracking

Finally, the model’s energy is also represented by the COT or metabolic cost ($40\%$). In this case, the model from Umberger et al. [125] is often used. Different metabolic cost models exist in the literature. Although they provide a good estimate of the measured metabolic cost, most models underestimate its value [126]. Additionally, the treatment of negative muscle work inside the models is still an open question [126]. The different representations of energy and effort are often combined inside the cost function (Fig. 5).

#### Task-specific goals

Half of the papers introduce task-specific goals, like reaching a maximal height with the centre of mass during jumping [127, 128] or maximizing the stride frequency for sprinting [129]. Additionally, $29\%$ encourages the model to move at a desired speed by adding a tracking term inside the cost function. The speed can also be enforced as a separate constraint, instead. The latter is often realised in OC solutions employing direct collocation [124].

#### Enforcing physiological limits

Other works penalize certain behaviours of the model to remain within physiologically realistic limits ($38\%$) or encourage stability ($37\%$). Overextension of the knee and ankle (ligament joint torques or joint angles), passive joint torques and impact forces with the ground are penalized for preventing unrealistically high values [90]. Large accelerations of the head or joints and jerk are also penalized to improve stability and to smoothen the motion [50, 54, 70]. Finally, some papers include penalties related to the contact with the floor [40, 71, 97] and the position of the centre of mass with respect to the base of support [54, 55, 71].

#### Tracking to improve predictions

Next, $36\%$ of papers include a term, tracking (experimental) data, inside the cost function. Works optimizing the muscle excitations use joint angle tracking, often combined with ground reaction force tracking [54, 73, 78] (Fig. 5). Papers optimizing model parameters also use pelvis pose data, angular velocities or even muscle excitations [79, 112, 116]. The tracking term aims to guide the solution towards a more human-like motion [80]. In total, $29\%$ of included papers use experimental data to predict the motion. However, some papers only use the experimental data to tune neural model parameters. The tuned model is in turn used to predict movement in new situations. As a result, $89\%$ of the included papers can predict motion without using experimental data or by creating a model that can predict motion in novel situations, for which no data was recorded. The distinction between tracking and prediction is further discussed in the “Discussion” section.

In total, $58\%$ of papers using DRL, include tracking terms. This is also called imitation learning. Tracking terms are included to ensure convergence, decrease the training time, increase training performance and obtain a natural walking pattern [80, 83]. Additionally, the training data allows the simulation of specific movements like deadlifts, cartwheels or kicks [18]. The training data consists of joint angle and joint angular velocities [14, 18, 78–80, 82], joint accelerations [14, 18, 87], pelvis pose and velocity tracking [14, 78–83] and end-effector pose, velocity or acceleration tracking [14, 18, 82, 87].

#### Encouraging movement

Finally, $30\%$ of papers include a term encouraging the model to keep moving. Penalizing falling is the most common example ($22\%$). This penalty is mainly used in works using DRL or an iterative optimization to tune their neural model.

## Musculoskeletal models

**Fig. 6:**
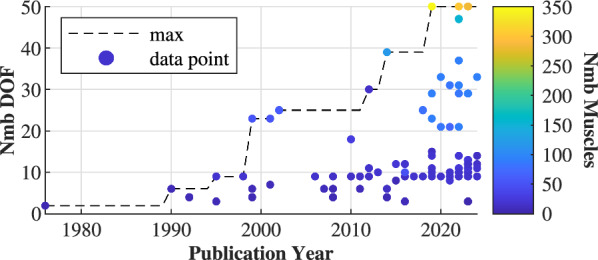
Evolution of the number of degrees of freedom (DOF) and muscles (colorbar) of the musculoskeletal models. The complexity of musculoskeletal models has increased significantly over time. Only papers for which both DOF and number of muscles are reported are visualized

MSK models predominantly represent adults ($97\%$), with only $5\%$ studying children. Three papers model both. MSK models are classified as either 2D ($73\%$), confined to the sagittal plane, or 3D ($27\%$). Models can reach up to 50 DOFs [14, 18, 88] and 700 muscles [122] (Fig. 6). Compared to the human body, which contains over 600 skeletal muscles, models typically ignore DOFs of the foot, arms, spine and hand joints.

2D MSK models typically comprise 7 rigid elements (foot, shank, tight and trunk), connected by 6 hinge joints (hip, knee, ankle) with a median number of 9 DOFs. The number of DOFs varies between 2 DOFs (right leg model) [8] and 15 DOFs (person with prosthetic leg) [69], incorporating between 4 to 48 muscles, with a median of 18. Su et al. [128] used a basic 2D MSK model with 3 DOFs and 4 muscles - the vasti muscle group, rectus femoris gluteus maximus and the hamstrings muscle group - to simulate jumping. Porsa et al. [63] used 48 muscles (24 per side), with a model based on the generic “gait2392” OpenSim model [130].

The 3D MSK models have between 10–50 DOFs (median of 25) and 16–700 muscles (median of 86). [44, 82] use a model of 8 internal DOFs (ankle, knee, hip and hip roll). Three studies use the DART simulation software to implement a model of 50 DOFs with 284 [14], 304 [88] and 346 [18] skeletal muscles. Wang et al. [97] use only 16 muscles (8 per leg). The muscles often include the SOL, GAS, TA, HAM, GLU, VAS and HFL. Zuo et al. [122] use 700 muscles and a DRL solution, with muscle synergies to reduce the problem’s dimensionality.

The majority of papers ($91\%$) use a Hill-type muscle model, while others either use a custom model [17] or do not represent the muscles on the skeletal structure [106, 107, 117], while still computing the generated muscle torque.

Ground interaction modelling is achieved through the Hunt-Crossley contact model ($46\%$), (nonlinear) spring-damper models ($25\%$) and Coulomb friction models ($4\%$). The ground contact is typically modelled by spheres, with a radius, stiffness and damping value. The foot model in predictive simulations is often simplified. Multi-segment foot models are only considered up to 3 segments, without considering a deformable foot arch [131].

Finally, model personalization is an important factor when designing person-specific devices or recommendations [25]. Only $28\%$ of studies adapted their MSK model to specific individuals. Of these, $91\%$ scale the model to the subject’s size, for example, by using the built-in OpenSim scaling tool, and $9\%$ adapts the muscle properties, like the optimal fibre lengths and tendon slack lengths [16] or the peak isometric force and tendon slack lengths [62]. Only $9\%$ tunes the ground contact parameters, i.e. the location of the contact spheres, their radius or stiffness [16, 47, 131]. While no papers explicitly adapt the MSK model to the subject’s sex, instead using one MSK model, based on either male or female physiology [97, 113], some aspects of sex-specific physiology are addressed through scaling. More research is required to uncover whether scaling sufficiently personalizes the MSK model.

## Accuracy & validation

### Validation strategies

To validate the predictive simulations, $81\%$ of papers compare their results to experimental data (Table 2). Of these, $92\%$ use kinematics data, like joint angles or joint angular velocities, $59\%$ use ground reaction force data and $57\%$ use electromyography recordings. Finally, $34\%$ of works use kinetics data, like joint moments and $12\%$ use metabolic cost measurements. Validation metrics include (Pearson) correlation coefficients, root-mean-square errors (RMSEs), symmetry indices [86] and qualitative comparisons. Only $50\%$ of papers performing validation, compute quantitative metrics. Other papers use mostly qualitative comparisons, based on figures overlapping simulation and experimental data. This is especially the case for EMG data (Table 2). Only $19\%$ of papers using EMG data, compute a quantitative metric, like a correlation coefficient. Since the metabolic cost is a single value, most papers compare it quantitatively to experimental measurements.

**Table 2:** Papers using experimental data for validation mostly use kinematics, ground reaction forces (GRFs) and EMG data. The cost of transport (COT) is less used for validation. Still, less than half compute quantitative metrics, especially when comparing EMG signals

	Kinematics (%)	GRFs (%)	EMG (%)	Kinetics (%)	COT (%)
Use validation data	92	59	57	34	12
Quantitative metric	46	24	11	16	11

Of the papers reporting the number of test subjects, $22\%$ use a single subject’s data, $58\%$ use ten or fewer test subjects, $33\%$ use between 11 and 50 participants, and only $9\%$ use data from more than 50 subjects. In terms of the subject’s sex: $93\%$ of papers disclosing the subject’s sex use male subjects, and $62\%$ use female subjects. Of these, $56\%$ uses both male and female subjects. Thus, $38\%$ uses only male subjects and only $7\%$ uses solely female subjects.

### Quantitative metrics

**Fig. 7:**
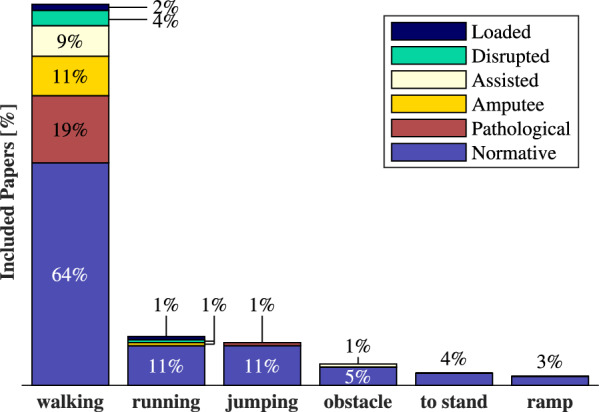
Movement types appearing in more than 2% of papers. Normative movement refers to healthy motion. Pathological movement introduces pathologies (e.g. muscle weakness). Works simulating a prosthesis or assistive device are denoted as “Amputee” or “Assisted”. Loaded and disrupted simulations add weights or disturbances to the model

Most of the reported quantitative metrics come from works using OC (18) and muscle-reflex (17) methods. Less information is available for RL methods (8) and CPGs (4). Detailed metrics can be found in the supplementary material. Here, we present the main conclusions for normal walking, as this is the most common simulated movement (Fig. 7) and allows for some comparison between methods.

OC solutions can reach errors smaller than the typical inter-subject variability [132] in kinematics during walking [47], especially when using inverse optimal control [71] or when including tracking terms [133]. Muscle-reflex models report RMSEs larger than one standard deviation, except for the ankle [93, 134], while DRL errors can reach even higher [80]. Most works reporting quantitative metrics show high correlations for the kinematics, except for the ankle in muscle-reflex-based methods [135, 136] and CPGs [108, 121].

Overall, methods struggle most at predicting ankle kinematics and non vertical GRFs. Most works are unable to reduce errors below intra-subject variability, which is partly due to the lack of personalized models, unless explicitly including experimental data.

## Applications & development

At first (1990–1999), jumping motion dominated the predictive simulations, for its straightforward objective: maximizing the center of mass’ height [2, 60, 127]. The works of Taga et al. [107], Anderson et al. [62] and Geyer and Herr [42] shifted the focus to walking prediction using CPGs, optimization and reflex-based methods respectively. Additionally, Pariser et al. [137] were the first to simulate treadmill gait in 2022, using OC. Finally, van den Bogert et al. [46] and Falisse et al. [49] improved the efficiency of OC problems by introducing DC and AD to OpenSim. Walking dominates the simulated movements ($72\%$), followed by running ($13\%$) and jumping ($12\%$) (Fig. 7). Additional simulations include navigating obstacles ($5\%$) [44, 96, 106], ramps ($4\%$) [44, 81, 91, 98] or sit- or squat-to-stand motion ($4\%$) [57, 60, 89]. Finally, two papers simulate sit-to-walk movements [100, 101].

Several papers consider pathological movement (Table 3) during walking ($21\%$) and jumping [138]. Reducing the muscle excitations is used to simulate neural weakness by using the gain parameters of the Geyer and Herr model [90], or to simulate stroke patients by reducing the neural excitations of a CPG model [17]. Spastic hyperreflexia is modelled by adapting the muscle-reflex model: by adding positive length and velocity feedback to the quadriceps (RF, VAS) and ankle plantar flexor muscles [90], or by increasing the muscle-reflex gains for the RF, HAM, GAS, SOL muscles [104]. Falisse et al. [16] model cerebral palsy by personalizing the tendon slack lengths and optimal fibre lengths. They also simulate spasticity through delayed feedback from the muscle-tendon force and its first-time derivative. Additionally, they identify personalized feedback gains, used in the muscle activation dynamics, minimizing the difference with EMG signals. Muscle weakness is simulated by reducing the maximal isometric force [15, 75, 88, 99], for example of the hip and ankle plantar flexors [39], the SOL [79, 134, 139], GAS [134, 139], GLU [109], TS and TA [140] muscles. Hemiplegic patients are simulated by reducing the maximal force on a single side of the body [14, 141]. Tightness in the muscles [75, 88] or muscle stiffening [18] is modelled by shortening the tendon slack length and optimal fibre lengths. Veerkamp et al. [105] model idiopathic toe walking by shifting the passive ankle moment-angle curves by $20^\circ$, and by adapting the optimal fiber and tendon slack lengths for the GAS and SOL muscles. Shachykov et al. [102, 112] use a higher-level control signal in their CPG model to prevent muscle excitation during a short time, to model freezing of the gait as in Parkinson patients. They control both the reflex and feedforward stimuli of the CPGs to model disruptions. Finally, some papers model skeletal pathologies by making one extremity shorter than the other [109], or twisting the femur geometry to model femoral anteversion [18].

**Table 3:** Summary of the simulated movement types

Type	Subtype	Sources
Pathologies	Neural weakness	[17, 90]
	Cerebral palsy	[16, 104]
	Muscle weakness	[14–16, 75, 79, 88, 99, 100, 109, 134, 138, 140, 141, 144, 145]
	Asymmetric leg length	[109]
	Muscle stiffening	[18]
	Parkinson	[102, 112]
	Toe walking	[105]
Prostheses	Transfemoral	[18, 43, 80]
	Transtibial	[18, 39, 46, 68, 69, 108, 109, 135, 142]
Assistive	Orthosis	[15, 79, 139, 145]
Devices	Ankle exo	[55]
	Hip exo	[123]
	Lower limb exo	[14, 78, 146, 147]
Disrupted	(Mechanical) force	[75, 82, 107]
	Changing terrain	[107, 119]
	Electrical stimulation	[95]
Loaded	–	[73, 107]

Prosthetic movement simulations ($11\%$ walking, $1\%$ running [18]) have shown the benefits of optimizing the prosthesis for a specific movement or MSK model [43, 142]. Some papers include the prosthesis parameters [68, 109, 142] or control signals [46, 69, 80] inside the optimization. However, some difficulties remain in transferring erratic force patterns to real prostheses [80, 143]. Studies have shown comparable COT between prosthetic and normal walking [39] for a passive transtibial prosthesis, though an increased energy expenditure in the remaining muscles [68]. Other works show the effect of walking with a prosthesis on energy expenditure [39] or gait kinematics [68, 109], and the need for amputees to adapt their gait pattern to reduce the joint reaction forces at the hip and knee [135] and their metabolic cost [108].

Assistive devices ($9\%$) are typically represented by ideal torque actuators [55, 146, 147] or CAD models with interaction forces [14, 79]. The devices are simplified by, for example, not considering their weight [15, 55, 79, 146] or by representing the interactions as fixed weld joints [78]. Additionally, some papers include the assistance profile or the device parameters (e.g. ankle orthosis stiffness) inside the motion prediction or optimization [14, 15, 55, 146].

Finally, $4\%$ and $2\%$ of papers simulate disrupted and loaded motion. Haeuffle et al. [119] found the importance of combined muscle-reflex and feedforward control strategies (i.e. CPGs) for antigravity muscles (SOL, VAS, GAS) to anticipate the changing ground level. Similarly, Song et al. [95] mentioned the need to tune muscle-reflex gains to improve the correspondence with experimental data. They mention CPGs as a way to gradually change the reflex gains between different parts of the motion. Anand et al. [82] present a model, trained using RL, that can withstand perturbations up to 200 Nm at the hip. However, the perturbation response is highly random. The nonlinear learned policy and passive muscle dynamics are given as possible explanations. Taga et al. [107] present a CPG-driven model that can regain steady state after several step cycles of applying a mechanical perturbation of 200 N to the HAT (head-arms-torso) segment. Their model can also generate stable walking on a slope of $\pm 2\%$ and walk with a load attached to the pelvis up to 15 kg. The main effects of this load are on the speed and step length. van den Bogert et al. [73] use OC to predict the effects of masses placed at the thigh, knee, shank and foot. They found an increased energy expenditure proportional to the mass, which was the largest for the foot or ankle.

Different methods suit various movements (Table 4): OC excels at movements with clear objectives, like walking, jumping, running and sit-or squat-to-stand movements. CPG and muscle-reflex-based models, although created for walking [42, 107], can also handle obstacles [44, 96, 106], ramps [44, 91, 98] and sit-to-stand [148] or sit-to-walk movements [100]. DRL can simulate diverse movements [18], like stair climbing [81], or deadlifts, kicking and cartwheels [18].

**Table 4:** Distribution of simulated movements for each method: optimal control (OC), muscle-reflex-based methods (reflex), deep reinforcement learning (DRL) and central pattern generators (CPG)

	Walking (%)	Running (%)	Jumping (%)	To-stand (%)	Obstacles (%)	Ramps (%)	Sit-to-walk (%)	Stairs (%)	Hopping (%)
OC	22	7	11%	2%	ND	ND	ND	ND	1%
Reflex	29	1	ND	1%	3%	3%	2%	ND	ND
DRL	14	4	2%	1%	3%	2%	ND	1%	1%
CPG	13	2	ND	ND	1%	ND	ND	ND	ND

*ND* no data available

## Discussion

In this final section, we discuss the results of the review process. The “How do we define predictive motion?” section gives an answer to the first research question by providing a definition of predictive movement. The “The limits of musculoskeletal models” section summarizes the main limitations of current MSK models. We discuss different approaches to generating predictive movement (Table 5) in the “Black Box vs. model-based neural controllers”, “What is the difference between DRL and OC?” and “Should we model the human neural controller?” sections. Finally, we evaluate current validation practices in the “Quantitative validation is lacking” section.

**Table 5:** Summary of predictive musculoskeletal simulations

Method $\rightarrow$	Black Box neural controller		Model-based neural controller	
Criteria $\downarrow$	Optimal control	Deep reinforcement learning	Muscle-reflex-based models	Central pattern generators
Simulation time	<1 h, days	> 1 h, days	NA	
Inputs	• MSK model state	• MSK model state	• Muscle state	• MLR input (opt.)
		• Interactions	• Trunk angle	• Proprioceptive feedback
				• Exteroceptive feedback
Outputs	• Muscle excitations	• Muscle excitations	Muscle excitations	
		• Joint accelerations		
Optimization variables	• Muscle excitations	• Neural network weights	• Reflex gains	• CPG parameters
	• Initial conditions		• Relfex delays	
	• Simulation time			
Optimization algorithms	• Direct collocation	• PPO		
	• Direct shooting	• DDPG	CMA-ES (tuning)	
		• PSA		
Cost function terms	• Muscle activations	• COT	• COT	• Overextension
	• Trunk excitations	• Falling	• Desired speed	• (Head) stability
	• COT	• Forward progression	• Falling	
	• Joint & GRF tracking	• Stability		
	• Overextension			
Experimental data	• Initial guess	• Imitation learning		
	• Improve human likeness	• Reduce simulation time	Only for tuning	
		• Improve human likeness		
Frameworks	• PredSim [39]	• OpenSim-RL [77]	• SCONE [10]	• Rybak [115]
	• OpenSim Moco [65]	• MyoSuite [13]	• Geyer and Herr [42]	

### How do we define predictive motion?

The definition of "predictive" motion lacks clarity in the literature, with terms like “forward”, “predictive” or “dynamic” being used interchangeably. Additionally, titles, abstracts and keywords are often insufficient to know whether the movement is truly predictive or just tracking experimental data. As a result, the inter-reader reliability metrics for the abstract screening are worse compared to the text screening phase.

In this paper, we defined papers as predictive in two situations. First, when it determines movement patterns independently of experimental data. This is the same definition as proposed by De Groote and Falisse [25]. However, we extend their definition to works using experimental data, depending on how the data is handled. When data is used to warm start a simulation (OC), or to tune neural model parameters (muscle-reflex, CPGs and DRL), we still consider the technique predictive. In these cases, the solution can make predictions in novel situations where no experimental data is available. However, these solutions are not independent of the initial guess and thus experimental data. Generally, it can be difficult to draw a line between tracking and prediction.

Overall, $18\%$ of works use experimental data to tune model parameters, like neural network weights or control gains, not counting papers that warm start the optimization problem with tracking solutions (e.g. OC [51, 141, 149]). While these models can technically be applied to new situations, they risk overfitting to the training data, and may extrapolate poorly to significantly different environments.

We classified $11\%$ of papers as semi-predictive, primarily OC works that include tracking terms directly into the objective function [68, 73, 142]. While tracking is not their only goal, explicitly including tracking terms in the cost function limits extrapolation potential, especially, since OC solutions output a time series of muscle excitations rather than controllers. Thus their ability to predict novel motion can be questioned. This also includes the work of Zhang et al. [114], who use experimental data to tune a CPG model, which explicitly uses data to adapt weight parameters.

### The limits of musculoskeletal models

#### OpenSim, the gold standard?

Most works use OpenSim models in OpenSim or SCONE to simulate the MSK system, as these are free and open-source. Additionally, validated OpenSim models and user codes are freely available on the SimTK website. Moreover, tools like OpenSim Moco [65], OpenSim-RL [77] and SCONE [10] allow users to explore OC, DRL and muscle-reflex-based solutions with their OpenSim models. Other software includes commercial options (AnyBody) and physics-based engines (Dart [14, 18, 87, 88], Simscape Multibody (MathWorks Inc.) [7, 95], SD/FAST [62, 127], Open Dynamics Engine (ODE) [40, 97] and MuJoCo [82, 136]), though these aren’t specifically designed for MSK modelling.

The NEURIPS 2017 challenge [77] and MyoChallenge (2022–2024, ongoing) have encouraged exploration of DRL solutions inside OpenSim and MuJoCo, which can simulate interaction forces when adding assistive devices to the MSK system [13]. Recently, MyoSim [150] and MyoSuite [13] were developed to bridge OpenSim models with MuJoCo and to provide a framework to use RL in MuJoCo. Another (commercial) alternative is HyFyDy, which combines the same MSK models as OpenSim, with MuJoCo’s speed.

Still, (OpenSim) models have some limitations, affecting the predicted motion. The MSK models simplify the soft tissue and joint behaviour [133], the Achilles tendon stiffness is too stiff in the basic OpenSim “gait2392” model [124, 131], and OpenSim provides limited support for interactions with external devices like exoskeletons [13]. Most works constrain the model to the sagittal plane (2D). These models are limiting, as there is considerable out-of-plane movement of the ankle and foot during walking [131]. Knee and hip joints are simplified by hinge joints [130, 135], or the torso is simplified, removing, for example, the arms. These simplifications affect the predicted motion and the model’s energy [15, 139]. The realism of muscle and ground contact models has remained largely the same over time, while the number of DOFs and muscles increases (Fig. 6).

#### Muscle models

The Hill-type model, used by almost all works to represent the muscles, and the default model in OpenSim, has some limitations. Muscle length changes and velocities are typically overestimated, due to the simplified geometry [93, 151], and the model poorly captures the stiffness and damping properties of actively lengthened muscles [47]. The Huxley Cross-bridge model, for example, is more accurate, especially for estimating the energetic cost [25]. However, it is also more computationally expensive. Thus, extensions to other muscle models could provide insightful additions to the literature.

#### Ground contact models

The ground contact is crucial to obtain accurate motion and GRF predictions [47], but is often a major limitation of the simulations [86]. The majority of papers use a Hunt–Crossley ground contact model, which is also implemented by default in OpenSim. Other works use similar concepts, to represent the ground contact, with spring-damper systems. The foot is considered rigid, with contact spheres typically placed at the toe and heel. Some works extend the foot model with a finite element representation of the foot tissue [133]. Additionally, modelling the toe joints can improve the knee kinematics, ankle and knee kinetics and reduce the vertical ground reaction force at impact [124, 131]. Finally, geometries, other than circles and spheres, like ellipses can also be used to represent the ground contact [47]. While the ground contact is a crucial part of the model’s interactions with the environment, it is often simplified and not calibrated. As a result, vertical ground reaction forces show large peaks at ground impact, while horizontal GRFs are difficult to predict accurately.

#### Personalization

Less than one-third of papers choose to personalize the MSK model (“Musculoskeletal models” section). Optimization, combined with a generic model, is often used to overcome the lack of personalization. The preferred method of personalization consists of scaling the model, which is a basic feature of MSK software like OpenSim. Tuning the ground contact or musculotendon parameters is far less common. Still, tuning the ground contact model significantly affects the predicted ground reaction forces [47]. The lack of experimental data makes it difficult to personalize musculotendon properties [152]. Falisse et al. [16] use both EMG and joint torque data to tune the muscle parameters. Anderson et al. [62] matched experimental torque-angle curves to their models. There is currently no general framework for the personalization of force-generating parameters within muscle-tendon units. Similarly, such framework is equally lacking for personalizing neural parameters. While some papers [102, 104] use kinematic data to tune the neural model parameters, further research is required into this aspect of personalization, as tuning the model to reproduce experiments, could reduce its predictive capabilities. Additionally, the MSK models are rarely adapted to the subject’s age or sex. Although some papers model children [40, 88, 104], the MSK model is simply scaled from a generic one. However, developing children show gait patterns that deviate from the typically used minimal effort objective [153]. Additionally, studies [154, 155] have shown sex-related differences in movements like walking and running. Moreover, there still exists a large disparity in male and female participants in biomechanics research [156]. Finally, while several works study pathological walking, other types of movements (e.g. jumping, running, avoiding obstacles) are rarely studied for patients, who also deal with these types of motion in everyday life.

The complexity of the MSK models is tightly linked to the computational capabilities of the available hardware and the convergence of the optimization algorithms. However, researchers should think about the required complexity of the MSK model, based on the proposed research questions. Increasing the model’s complexity may not result in a marginal increase in accuracy or alter the conclusion [152]. Yet, studies rarely include convergence studies to test this hypothesis. D’hondt et al. [131] and Falisse et al. [124] are the only exception, ensuring that the simulations are not impacted by a finer mesh and tighter convergence tolerance, or the choice of the initial guess.

### Black Box vs. model-based neural controllers

Model-based neural controllers (muscle-reflex, CPG) struggle to fully capture human complexity, particularly the integration of anticipation and higher-level thinking is still lacking in these models. Human walking, for example, is driven by more than just muscle-reflexes or feedforward signals. Combinations of both models are an interesting avenue for future research.

Black box neural controllers (DRL, OC) rely on optimization to overcome the lack of a mechanist model, making it crucial to define the right objective function. While most works employ a linear combination of cost function terms, experimentation with multi-objective optimization is scarce, except for the use of bilevel optimization, to tune weighting factors [54, 70, 71]. However, it remains an open question how the optimization weights should be selected. Moreover, like the gait goals, these weights are also subject-dependent.

While OC captures many of the salient features of walking, it is unknown whether objective optimization truly captures the human neural controller. The basic linear combinations still entail some limitations [70] and it is unknown whether additional terms (Fig. 4) are missing.

The assumption of minimal energy consumption may not hold for certain populations [153], such as patients with neuromuscular disorders, who might prioritize factors like pain reduction over effort, or children. Still, several papers use optimization, even when dealing with pathologies [79, 141] or children [16, 88]. Additionally, in some cases, like in Parkinson’s disease, it is unknown which neurological adaptations are required to explain the pathological motion. Furthermore, it is difficult to know whether errors are related to MSK modelling, the neural controller or the optimization objectives. Finally, many works consider their results to be local, rather than global optima [139].

Two papers combine both model-based controllers with DRL. We highlight an important distinction between RL and model-based parameter tuning. Both approaches iteratively improve the performance by tuning the parameters of the controller, based on a reward or fitness function. Model-based tuning, particularly using CMA-ES with CPG and muscle-reflex modules, use a well-defined mathematical model rather than a black box controller. However, CMA-ES evaluates the performance with a fixed controller, making the method static. In constrast, RL continously adapts its controller during evaluation. Su et al. [45] uniquely use CMA-ES within a RL context, to tune a muscle-reflex-based model.

### What is the difference between DRL and OC?

In terms of their goal, OC and DRL are not fundamentally different, as both methods aim to optimize an objective function. However, they use different approaches. OC determines the time series of unknown muscle excitations, while DRL optimizes network weights, defining a control policy. Neither method provides much insights into real life neural control mechanisms. The neural network in DRL is still an abstract representation of the human neural controller. Additionally, the objective terms are sensitive to the chosen method (Fig. 4). DRL typically requires additional rewards to converge beyond effort minimization, for example, also encouraging the model to keep walking. For OC, minimizing effort is often already sufficient [141].

In OC, even though the same muscle excitations can be used to drive a forward simulation in a new environment, the model will not adapt its movement to this new situation. Instead, the optimization should be re-run to account for these changes, making it less suitable for dynamic scenarios like unexpected obstacles. Additionally, the black box nature of the solution complicates modelling pathologies through neural parameter adjustments. In contrast, model-based neural controllers and DRL produce adaptable policies, allowing to change feedback parameters [92], CPG variables [17] or higher-level inputs [102, 112], even in real-time [88]. However, DRL requires a comprehensive training process to ensure extrapolation to new situations [79].

OC can generate realistic movement patterns, of walking, running, jumping and sit-to-stand or squat-to-stand movements. Additionally, by applying algorithms like direct collocation, combined with algorithmic differentiation and implicit differential equations, the simulation time of OC solutions has been reduced to 36 min [49] or less. This is significantly lower compared to the lengthy training times of 1 h [86] to several days [88], reported for DRL or the required time of 2 h [40] or days [109] to optimize model parameters for muscle-reflex-based and CPG models. However, the simulation time also largely depends on the available hardware.

DRL, although quite recent, shows promising results [80, 86], but faces challenges to produce human-like solutions [80, 84]. While experimental data is not strictly required to obtain realistic results [88], it is often an essential part of many DRL solutions, to improve the human-likeness of the solutions but also to ensure convergence, decrease the training time and increase the training performance. Hence, DRL works using a more detailed model, in 3D [82, 83] and using a large number of muscles ($> 100$) [14, 18, 87] often resort to experimental data. While DRL solutions offer many innovations in machine learning, they often lack critical validation of biomechanical aspects, like joint impedance or co-activation patterns of muscles. This strongly limits their use in clinical settings.

### Should we model the human neural controller?

Opposite to OC solutions, or the neural networks used in DRL, muscle-reflex models provide a mathematical model of the proprioceptive muscle reflexes, allowing researchers to study different (neural) pathologies [15, 99, 112], by changing the muscle activation levels [90], adding additional feedback laws [90] or by changing feedback parameters [104]. However, the muscle-reflex-based model of Geyer and Herr was mainly designed to generate walking. Moreover, human walking is not only driven by reflexes. While extensions of the model have been applied to running, ramps, navigating obstacles, adaptations to disturbances [95, 119] and sit-to-walk movements [100], the range of movement is limited. The same model cannot be used to predict jumping motions or stair ascent/descent, and they struggle to extrapolate, compared to DRL, when the motion deviates a lot from normal walking [44]. Finally, we excluded works predicting standing or dealing exclusively with posture control. Instead, we refer the reader to the review of Shanbhag et al. [26] on this topic. While these cases appear more simple compared to, for example, predicting gait, they can still teach us critical aspects of balance, which are essential for more complex movements.

Muscle-reflex-based methods show high correlations for the hip and knee during walking, but lower values for the ankle joint, especially during the swing phase of walking [42]. The absence of modelled toes could affect the timing of the swing phase and hence the ankle joint angle correlations [118].

CPGs take on different shapes in the literature, making them more difficult to classify and describe. Additionally, they often combine several concepts, like proprio- and exteroceptive feedback [116], muscle reflex modules [118, 119], balance controllers [116] and muscle synergies [121]. These synergies are relatively unexplored inside the literature, as only five papers use them. However, the synergies could be of great value in reducing the parameter space and unknown variables, allowing researchers to simulate more complex models. Similar to the muscle-reflex-based models, CPGs are designed to simulate walking movements, and are also used to study running and obstacle avoidance. Additionally, the detailed model allows researchers to make specific changes to study, for example, pathologies [112] and the effect of disturbances on the walking pattern [107, 119]. Validation results for CPG models are scarce, yet reported correlations (supplementary material) show high accuracies for the hip and knee, and lower values for the ankle [108].

### Quantitative validation is lacking

#### Simulation accuracies

Current validation practices are insufficient to establish meaningful comparisons between methods and works. The lack of available metrics, the variety in their units and the lack of a common benchmark dataset makes it difficult to compare accuracies between methods. While the correlation metric provides a relatively unambiguous result, the RMSE is expressed in both degrees and units of standard deviations, thus strongly depending on the used dataset. Some trends, like the worse correlations of the ankle joints, are shared among most methods. While the reflex-based methods consistently use correlation metrics, papers using DRL and CPG solutions lack thorough validation.

Common issues across methods include lower correlations of the ankle joint angle, specifically during swing. These are mostly related to the foot not being in contact with the ground [81]. Sufficient toe clearance is important, to prevent the model from stumbling or hitting an obstacle. Additionally, the lack of metatarsal joints also affects the ankle plantarflexion [49, 118]. For DRL models, toe-walking is another commonly observed issue, related to the model aiming to generate sufficient foot-ground clearance [86]. The lack of modelled foot muscles can be another reason, as the missing muscles are not penalized for being activated. Thus, improving the foot model could aid in resolving this issue [131]. Another frequently observed issue is the lack of knee extension during mid-stance, resulting in lower knee joint torques [39, 49].

For the GRFs, the simple trunk model, which is considered rigid, leads to higher impact forces at ground impact [7, 49]. While predictions of the vertical GRFs show high correlations ($R> 0.97$), the shear forces are often less accurate, due to simplified ground contact models, usually based on (nonlinear) spring-dampers.

EMG comparisons remain largely qualitative, due to inter-subject variability, difficult scaling and unknown musculotendon parameters, like optimum tendon lengths and maximal muscle forces [152]. However, the GAS, SOL and GLU muscles show consistently high correlations, although, the results are highly dependent on the model and method.

#### Recommendations

Lund et al. [28] provided recommendations on validating MSK simulations. Based on our results, these have yet to find their way into the general literature. Current validation practices have five main limitations.

Firstly, current papers largely focus on the prediction of normal walking (Fig. 7). To validate the predictive quality of the methods, there is a need to validate different gait conditions, as current errors can still be larger than inter-subject variability in experimental data.

Secondly, blind validation is lacking, as the simulation is often validated on the same data used to train or optimize the model [81–83]. For predictive simulations, it is crucial to assess the ability of the model to extrapolate to unseen cases, to have a meaningful discussion about their potential.

Thirdly, more than $80\%$ of papers include validation, but half of these are purely visual. Quantitative validation is not always straightforward, especially when dealing with EMG signals. Nevertheless, without consistent metrics, comparisons between methods are difficult.

Fourthly, the number of test subjects is often limited and in more than half of the included papers, less than 10. The diversity of the subjects is equally limited, with most works using male participants. Additionally, most works focus on (healthy) adults. Children and especially older adults are greatly under-represented in the data.

Finally, competitions, like the NEURIPS learning to run challenge [77] and the MyoChallenge [157], push researchers to benchmark predictive solutions. However, the submitted controllers are only evaluated on the travelled distance, not their human likeness, which plays an important role in many applications.

Additionally, only $22\%$ of works share their codes, limiting cross-validation opportunities and forcing novel users to build their own solutions or start from a limited set of available ones. By open-sourcing the model files and codes, important information, like muscle properties, ground contact models, optimization settings and more, can be retrieved and used to build a more comprehensive comparison.

## Conclusion

We identified four main methods for predictive musculoskeletal simulations: OC, DRL, CPGs and muscle-reflex-based models. OC solutions are accurate and efficient, but limited to optimal movements. Muscle-reflex-based models correlate well with experimental data for walking-like motions. CPGs show great variability in their implementation and can help study pathologies and disturbances. Finally, DRL can generate diverse movements but often needs experimental training data to produce human-like motion. Additionally, most works use optimization to find unknown muscle excitations or to optimize neural model parameters.

MSK models show increasing complexity, with more muscles and DOFs. Yet most are simulated inside of OpenSim, simplify the ground contact interactions and torso, and lack personalization, beyond scaling.

Quantitative validation remains a major limitation, with most works relying on qualitative comparisons and limited datasets. We recommend using consistent quantitative metrics, based on benchmarking datasets, and open-sourcing model files and codes, to properly compare methods.

Finally, we provided a definition of predictive MSK simulations, as the field needs clearer terminology to distinguish from works tracking experimental data.

## Supplementary Information

Supplementary Material 1. This file describes additional details of the review process, like inclusion criteria, search queries and data collection.

Supplementary Material 2. This file contains the main data entries collected for each paper.

Supplementary Material 3. This file summarizes additional results from the review paper, including a table of metrics for each method.

## Data Availability

Data is provided within the manuscript or supplementary information files.

## Abbreviations

OC: Optimal control
NLP: Nonlinear programming
DC: Direct collocation
DS: Direct shooting
AD: Algorithmic differentiation
(D)RL: (Deep) reinforcement learning
CL: Curriculum learning
CPG: Central pattern generator
RG: Rhythm generator
PF: Pattern formation
MN: Motor neuron
RMSE: Root-mean-square error
EMG: Electromyography
DOF: Degree of freedom
GRF: Ground reaction force
COT: Cost of transport
MSK: Musculoskeletal
NMSK: Neuromusculoskeletal
GAS: Gastrocnemius
HFL: Hip flexor
SOL: Soleus
GLU: Gluteus
VAS: Vasti
TS: Triceps sural
RF: Rectus femoris
HAM: Hamstrings
TA: Tibialis anterior
