# Supplementary material for: A PRISMA systematic review through time on predictive musculoskeletal simulations
Source: J Neuroeng Rehabil. 2025 Jul 4;22:149. doi: 10.1186/s12984-025-01686-w (PMC12228224; doi:10.1186/s12984-025-01686-w)
Supplement: Supplementary file 1 — Supplementary Material 1. This file describes additional details of the review process, like inclusion criteria, search queries and data collection. [file 12984_2025_1686_MOESM1_ESM.pdf]

# A PRISMA systematic review on predictive musculoskeletal simulations

# Authors

## Contact

BruBotics
Vrije Universiteit Brussel
Pleinlaan 2,
1050 Brussels
Belgium

# Contributions

| Name | Affiliation | Email | CRediT Contribution |
|---|---|---|---|
| Menthy Denayer | BruBotics, Vrije Universiteit Brussel and Flanders Make | menthy.denayer@vub.be | Conceptualization, Formal analysis, Investigation, Resources, Data curation, Writing - Original Draft, Writing - Review & Editing, Visualization, Project administration, Funding acquisition |
| María Alejandra Díaz | MFYS, BruBotics, Vrije Universiteit Brussel | ma.diaz@vub.be | Writing - Review & Editing, Conceptualisation, Methodology, Validation, Investigation, Data Curation, Visualization |
| Eligia Alfio | MFYS, BruBotics, Vrije Universiteit Brussel | eligia.alfio@vub.be | Writing – Review & Editing, Conceptualisation, Methodology, Validation, Investigation, Data Curation, Visualization |
| Tom Verstraten | BruBotics, Vrije Universiteit Brussel and imec | tom.verstraten@vub.be | Writing - Review & Editing, Conceptualisation, Resources, Funding acquisition, Supervision |
| Kevin De Pauw | MFYS, BruBotics, Vrije Universiteit Brussel | kevin.de.pauw@vub.be | Writing - Review & Editing, Conceptualisation, Resources, Funding acquisition, Supervision |
| Massimo Sartori | Neuromuscular Robotics research group, University of Twente | m.sartori@utwente.nl | Writing – Review & Editing |
| Friedl De Groote | Human Movement Biomechanics re-search group, KU Leuven | friedl.degroote@kuleuven.be | Writing – Review & Editing |

# Amendments

Any amendments to this document will be reported below:

| Last updated | Note |
|---|---|
| 03.03.2024 | Creation of the protocol for the systematic review. |

# Support

This work was supported by BOSA FOD AI-Driven Wearable Robotics for Healthcare (Aidwear). Menthy Denayer is a doctoral fellow of the Research Foundation - Flanders (FWO).

# Introduction

## Rationale

The present systematic review aims to give a comprehensive overview and classification of existing predictive simulations combined with musculoskeletal models to predict novel motions.

Predictive simulations are shaping up to be an important tool in the design of assistive devices (exoskeletons, prostheses), in studying human motion and the human neural controller, or in predicting the effect of surgeries and robotic devices on the human body.

Existing reviews on predictive musculoskeletal simulations are typically limited to a subdomain of solutions (optimal control, reinforcement learning), do not mention the human likeness of the simulations (validation), and fail to adhere to existing PRISMA guidelines (no mention of research questions, number of screened papers or ex-inclusion criteria).

To the best of our knowledge, the following reviews exist concerning the topic of (predictive) musculoskeletal simulations:

On the use of predictive simulations in character animation: [1]

On the use of deep reinforcement learning: [2–4]

On the use of (optimal) control solutions: [5–10]

On the application of predictive simulations: [11–13]

On MSK validation: [14]

## Objectives

The objectives of this review have been formulated with the PCC framework, and we are replacing Population with Problem, as often done for PICO [15].

| | | |
|---|---|---|
| **P** | **Problem** | Predicting human motion |
| **C** | **Concept** | Musculoskeletal models combined with optimization-based (including reinforcement learning) methods and control |
| **C** | **Context** | Surgery outcome prediction, effect and design of assistive devices (exoskeletons, prostheses) |

**The research questions the review will be addressing are:**

General overview:

**Q1:** How can we define movement prediction of a multibody, musculoskeletal system?

**Q2:** What are the limitations of current MSK models?

**Q3:** How can we generate predictive motion?

**Q4:** What are current validation practices?

# Methods

## Eligibility criteria

### Phase 1: Identification inclusion criteria:

We have defined the following criteria for **inclusion** in the first phase of the screening

| | |
|---|---|
| **Scientific Papers** | • The paper is written in English or an English translation is available<br>• The paper is a journal article or conference proceeding<br>• The paper is not a duplicate |

### Phase 2: Overview Screening inclusion criteria

We have defined the following general criteria for **inclusion** in the second phase of the high-level overview screening.

| | |
|---|---|
| **Scientific Papers** (Title, Abstract, Keywords) | • The paper is not a review paper<br>• The paper seems to focus on musculoskeletal models<br>• The paper seems to propose an algorithm which allows for the prediction of human motion<br>• The paper does not limit itself to tracking experimental data |

Exclusion and inclusion criteria for the **title-abstract screening** in Rayyan:

| Include | Exclude |
|---|---|
| **MODEL** | |
| What kind of model must be used? Who should the model represent?<br><br><u>Simulation Models</u><br>- musculoskeletal models<br>- neuromuscular models<br>- neuromechanical models<br>- muscle-torque generators<br><br><u>Model Representation</u><br>- adult or child<br>- full-body<br>- lower-limb | *What kind of model must be used? Who should the model represent?*<br><br><u>Simulation Models</u><br>- torque-driven models<br>- models lacking muscles (e.g. spring-mass models, pendulum models)<br>- avatars or characters<br>- biological models (organs, fluids, finite elements, soft tissues etc.)<br><br><u>Model Representation</u><br>- animals<br>- robots<br>- non-human models<br>- upper-body-only models |
| **MOTION** | |
| <u>Motion</u><br>- gait, locomotion, running, hopping<br>- arm swaying<br>- impaired/pathological/healthy/normal/assisted | <u>Motion</u><br>- jaw/eye motion<br>- arm reaching/hand motion/steering<br>- static balance control/posture control<br>- trunk motion, rowing |

| | |
|---|---|
| - sit-to-stand movement | - upper body movement without being part of lower body stabilization (e.g. arm swaying included, simple lifting not)<br>- outside of clinical usage (e.g. car ingress)<br>- inside earth's gravitational field |
| **SIMULATION TYPE** | |
| *What will we do with the model?*<br><br><u>Simulation Types</u><br>- **predictive** simulations (using no experimental kinematics, muscle activations, torques of forces) of motion/stance using the MSK model<br>- **semi-predictive** simulations (using imitation-learning to enhance simulation results, however, tracking is not only term in cost function) of motion/stance using the MSK model<br><u>Other</u><br>- optimization/control of assistive devices (exoskeleton, prostheses, etc.) **including** predictive simulations of the MSK model | *What will we do with the model?*<br><br><u>Simulation Types</u><br>- **no simulation**, experimental analysis<br>- musculoskeletal simulation focussed on the **analysis** of experimentally captured motion (e.g. tracking, cf. C)<br>- musculoskeletal simulations focussed on the prediction of physiological values, but not motion (e.g. contact forces, muscle forces, mechanical loads etc.)<br>- **model design** (no focus on predictive motion)<br><u>Other</u><br>- optimization/control of assistive devices (exoskeleton, prostheses, etc.) **without** predictive simulations of the MSK model<br>- functional electrical stimulation<br>- simulation of biological models (not musculoskeletal motion/stance) |
| **METHODOLOGY** | |
| *How do we achieve the motion/states prediction?*<br><br><u>Methodology</u><br>- muscle reflex models<br>- PID controllers<br>- neural networks<br>- machine learning<br>- optimal control<br>- gait models<br>- … | *How do we achieve the motion/states prediction?*<br><br><u>Methodology</u><br>- EMG data-driven models<br>- models are tracking experimental data (e.g. inverse dynamics/kinematics)<br>- Models are following predefined joint angle trajectories<br>- papers solving/improving the muscle redundancy problem with fixed kinematics (static optimization, CMC) |

## Phase 3: Full-text screening inclusion criteria

We have defined the following criteria for **inclusion** in the third phase of the full-text overview screening. The beforementioned criteria are still applicable, with the following clarifications:

| Include | Exclude |
|---|---|
| **MOTION** | |
| The paper considers (at least) lower limb motions, like gait, running, hopping, jumping, stair or ramp climbing (daily activities). The motion can be normative, assisted, pathological or concerning amputees. | Papers targeting motions that do not require a predictive element (e.g. cycling) or highly specific movements (e.g. skiing) are excluded. |

| | |
|---|---|
| For the motions, a healthy human should be able to perform the motions without the use of any external device. | Movements requiring the aid of an external device for a healthy human are excluded (e.g. skiing, cycling, pedalling etc.). |
| **MODEL** | |
| The paper uses a musculoskeletal model, i.e. a model driven by muscles (e.g. Hill-type), of a human (child, adult, elder), of at least the lower limbs. | Papers using torque-driven models, avatars or non-musculoskeletal models are excluded. |
| **METHOD** | |
| Papers predicting motion by optimization, control, learning or bio-inspired models. | Papers applying pre-defined signals (e.g. muscle excitations), without any physical basis are also excluded. |
| Tracking data in any shape of cost function is only allowed if it is not the sole term constituting the objective and under the condition that the tracking weight is not significantly higher compared to non-tracking terms (i.e., $w_{track} > 1000 \cdot w_{other}$). | Papers that are solely tracking experimental data in any shape or form are excluded. |
| **GOAL**<br>***at least 1 inclusion must be satisfied*** | |
| The paper develops a motion controller to predict novel movements. | Papers presenting software without a focus on predictive simulations are excluded. Even though the software might allow for such simulations (e.g. OpenSim), the paper itself must also discuss them in sufficient detail (i.e. not solely as a potential application).<br><br>The paper focusses on building a biomechanical or musculoskeletal model, instead of its neural controller. |
| The paper uses predictive tools (pre-existing or not) to study the influence of prostheses, pathologies or assistive devices on movement. | Papers focussing solely on analysing or segmenting experimental data are excluded.<br><br>Papers re-using existing predictive simulations, without discussing kinematic changes or effects are excluded. |
| The paper uses predictive tools (pre-existing or not) to develop a controller for a prosthesis or assistive device. | Papers designing devices, without considering predictive motion are excluded.<br><br>Papers re-using existing predictive simulations, without discussing kinematic changes or effects are excluded. |

# Information Sources:

## Scientific Papers:

**SCOPUS**, **Web of Science** and **PubMed** have been selected because they are some of the world's leading and competing abstract and citation databases for scientific literature. The identified papers are exported as RIS file from SCOPUS and Web of Science, and as an nbib file from PubMed. Exported papers are imported into Zotero to remove duplicates. The Rayyan tool is used for title-screening and the PICO platform for title-abstract and full-text screening.

| Source type | Source name | Publisher | Date last searched (DD.MM.YYYY) |
|---|---|---|---|
| Database | SCOPUS | Elsevier | 23.12.2024 |
| Database | Web of Science (WoS) | Clarivate | 23.12.2024 |
| Database | PubMed | United States National Library of Medicine | 23.12.2024 |

# Search Strategy:

## Scientific Papers:

| Data Base | Search String |
|---|---|
| SCOPUS | TITLE-ABS-KEY ( ( MUSCULOSKEL* OR MUSCULO-SKEL* OR NEUROMECH* OR NEUROMUSC* ) AND<br><br>( SIMULAT* OR "MUSCLE?REFLEX MODEL*" OR FRAMEWORK ) AND<br><br>( ( PREDICT* OR MIMIC* OR SYNTHESI* OR IMITAT* OR REPLICAT* OR REPRODUC* OR PRODUCE OR LEARN* OR GENERAT* ) W/10<br><br>( MOTION* OR MOV* OR LOCOMOTION OR GAIT OR ASCEN* OR DESCEN* OR "MOTOR SKILL*" OR KINEMATIC* ) ) AND<br><br>( OPTIM* OR MINIM* OR "STAB*" OR STEADY OR "SYMMETR*" OR HEALTHY OR IMPAIRED OR PATHOLOGIC* OR ASSISTED ) AND ( TRAIN* OR CONTROL OR FEEDBACK* OR REINFORCEMENT OR FORWARD OR DYNAMIC ) AND<br><br>( HUMAN* OR PERSON* OR SUBJECT* OR BIPED* ) AND NOT<br><br>( INSECT* OR FLUID* OR "LUNG*" OR "EYE*" OR "FUNCTIONAL ELECTRICAL STIMULATION" ) ) AND<br><br>PUBYEAR < 2024 AND ( LIMIT-TO ( DOCTYPE , "AR" ) OR LIMIT-TO ( DOCTYPE , "CP" ) ) AND<br><br>( LIMIT-TO ( LANGUAGE , "ENGLISH" ) ) |
| Web of Science | TS = ( ( "musculoskel*" OR "musculo-skel*" OR "neuromech*" OR "neuromusc*") AND<br><br>( "simulat*" OR "muscle?reflex model*" OR "framework") AND<br><br>("predict*" OR "learn*" OR "mimic*" OR "synthesi*" OR "imitat*" OR "replicat*" OR "reproduc*" OR "produce" OR "generat*" ) AND<br><br>( "motion*" OR "move*" OR "locomotion" OR "gait" OR "ascen*" OR "descen*" OR "motor skill*" OR "kinematic*") AND |

| | |
|---|---|
| | ("optim*" OR "minim*" OR  "stab*" OR "steady" OR "symmetr*" OR "healthy" OR "impaired" OR "pathologic*" OR "assisted") AND<br><br>("train*" OR "control" OR "feedback*" OR "reinforcement" OR "forward" OR "dynamic") AND<br><br>( "human*" OR "subject*" OR "biped*" ) NOT<br><br>( "insect*" OR "fluid*" OR "lung*" OR "eye*" OR "Functional Electrical Stimulation") )<br><br>AND DOP=(1000-01-01/2023-12-31) AND<br><br>LA = (ENGLISH) AND DT=(Article) |
| PubMed | ( "musculoskel*"[Title/Abstract] OR "musculo-skel*"[Title/Abstract] OR "neuromech*"[Title/Abstract] OR "neuromusc*"[Title/Abstract]) AND<br><br>( "simulat*"[Title/Abstract] OR "muscle?reflex model*"[Title/Abstract] OR "gait model*"[Title/Abstract] OR "framework"[Title/Abstract]) AND<br><br>("predict*"[Title/Abstract] OR "mimic*"[Title/Abstract] OR "synthesi*"[Title/Abstract] OR "imitat*"[Title/Abstract] OR "learn*" OR "replicat*"[Title/Abstract] OR "produce" OR "reproduc*"[Title/Abstract] OR "generat*"[Title/Abstract]) AND<br><br>( "motion*"[Title/Abstract] OR "move*"[Title/Abstract] OR "locomotion"[Title/Abstract] OR "gait"[Title/Abstract] OR "ascen*"[Title/Abstract] OR "descen*"[Title/Abstract] OR "motor skill*"[Title/Abstract] OR "kinematic*"[Title/Abstract]) AND<br><br>("optim*"[Title/Abstract] OR "minim*"[Title/Abstract] OR "stab*"[Title/Abstract] OR "steady"[Title/Abstract] OR "symmetr*"[Title/Abstract] OR "healthy"[Title/Abstract] OR "impaired"[Title/Abstract] OR "pathologic*"[Title/Abstract] OR "assisted"[Title/Abstract]) AND<br><br>("train*"[Title/Abstract] OR "control"[Title/Abstract] OR "feedback*"[Title/Abstract] OR "reinforcement"[Title/Abstract] OR "forward"[Title/Abstract] OR "dynamic"[Title/Abstract]) AND |

| | |
|---|---|
| | ( "human*"[Title/Abstract] OR "subject*"[Title/Abstract] OR "biped*" [Title/Abstract]) NOT<br><br>( "insect*"[Title/Abstract] OR "fluid*"[Title/Abstract] OR "lung*"[Title/Abstract] OR "eye*"[Title/Abstract] OR "Functional Electrical Stimulation"[Title/Abstract])<br><br>AND 1000/01/01:2023/12/31[Date - Publication] AND<br><br>"English"[Language] |

# Study Records

## Data management

The initial title-screening and abstract-screening is conducted in the Rayyan application. Full-text screening, segmentation and data extraction, are conducted in a custom Excel spreadsheet.

## Selection process

The identified papers are first imported into Zotero and afterwards included into Rayyan. The initial title-screening phase is conducted by a single reviewer, Menthy Denayer.

Afterwards, abstracts are screened in a blinded manner by two reviewers, María Alejandra Diaz and Menthy Denayer. After the blind screening, conflicts are re-evaluated by both reviewers in an unblinded way. The remaining conflicts are discussed until a consensus on their inclusion or exclusion is reached.

Full-text screening is performed in an unblinded way by 3 reviewers, María Alejandra Diaz, Menthy Denayer and Eligia Alfio. The reviewers must consent to a concrete reason for exclusion in the full-text screening phase.

We will be reporting the intra-rater reliability IRR data and Cohens Kappa consent coefficients after and up to the completion of the full-text screening phase.

## Data collection process

Data collection will be conducted by the **3** reviewers **María Alejandra Diaz**, **Menthy Denayer** and **Eligia Alfio** independently in Excel. Each reviewer performs the extraction independently and produces their own set of data. After each reviewer has extracted the document’s data, both reviewers jointly review both data sets together and extract data with consensus.

# Data Items

We will be extracting data items in PICO during the full-text screening. A full data extraction template can be found in **table 1**. We set ourselves free to update the data extraction template with additional categories throughout the review and inform about this with a protocol update and update the previously filled out data extraction forms.

**Table 1: Data Extraction**

| Field# | Field Name | Description | Options |
|---|---|---|---|
| **General Information** | | | |
| | **Title** | Give the full title of the document. | |
| | **Year published** | The year in which the document was published. | |
| | **Journal** | The journal in which the document was published. | |
| | **Q-value** | Q-ranking of the journal in which the document was published. | Taken from the Scimago database. |
| | **Research Group/Institution** | Group or institution where the work was conducted. | Multiple groups can be included using the separation character ';'. |
| | **PDF Retrieved** | Is a pdf version of the manuscript available? | • Yes<br>• No |
| | **Included in Identification** | Was this paper found based on the SCOPUS, WoS or PubMed search? | • Yes<br>• No |
| | **Description** | Describe the main goal of the paper. | This can include a novelty regarding motion prediction or a specific use case with predictive simulations. |
| **Methodology** | | | |
| | **Control Paradigm** | What is the key concept behind the proposed method to achieve predictive motion? | Options can include:<br>• objective optimization: e.g. minimizing effort, maximizing travelled distance etc.<br>• synergy-based: muscles are controlled as a combination of a small number of basis functions<br>• muscle-reflex-based: a control law (e.g. PID) is used to find the muscle excitations based on observed states<br>• state machines: different controllers are used depending on the state of the model<br><br>Multiple techniques can be included using the separation character ';'. |
| | **Method** | What is the main method (high-level) used to achieve this proposed concept? | Options can include:<br>• reinforcement learning: uses a basic controller to map the states to the inputs.<br>• deep reinforcement learning: uses a neural network to map the states to the inputs.<br>• optimal control: optimal control inputs are found by optimizing an objective function.<br>• PID control: using PID controllers or variants of feedback controllers.<br>• Biological Models: (differential) equations modelling biological modules |

| | | |
|---|---|---|
| | | Multiple techniques can be included using the separation character ';'. |
| **Algorithm/Model/Controller** | Specific algorithm or technique used inside the paper. | Popular options include: CMA-ES, PPO, direct collocation, DDPG/SAC, multiple shooting etc.<br><br>Multiple techniques can be included using the separation character ';'. |
| **Optimization Variables** | If applicable, which variables are optimized in the paper? | Popular ones include muscle excitations, initial states, control law parameters etc.<br><br>Multiple variables can be included using the separation character ';'. |
| **Cost Function Terms** | What are the terms used inside the cost function? | Short description of the term.<br><br>Multiple techniques can be included using the separation character ';'. |
| **Cost Function Shape** | What is the shape of the cost function? | Can be a linear combination, a single term, exponential terms etc.<br><br>Multiple techniques can be included using the separation character ';'. |
| **Imitation Learning** | Does the paper use reference data (e.g. inside a cost function) to improve the motion prediction? | • Yes<br>• No |
| **Predictive Quality** | What is the predictive quality of the paper?<br><br>*All papers fitting the "full tracking" category should be excluded.* | • Full Tracking: only tracking reference data.<br>• Semi-Predictive: predictive, with tracking elements.<br>• Fully Predictive: without using any reference data to predict the control input or when determining a policy that can predict motion without any reference data. |
| **Validated** | Does the paper compare the simulation results against experimentally measured data?<br>I.e. EMG data, kinematic data, GRF data. | • Yes<br>• No |
| **Quantitative Validation** | Is the method validated using quantitative metrics? | • Yes<br>• No |
| **Number of Subjects** | How much subjects were used to validate the simulation? | Number. |
| **Validation Data** | What type of data does the paper use to validate their simulation? | • EMG<br>• Kinematics: IMU-based or optical marker data.<br>• Kinetics: joint torques<br>• Ground Reaction Forces<br>• Metabolic cost: energy expenditure<br>• NA: if no validation is performed.<br>Multiple techniques can be included using the separation character ';'. |
| **Quantitative Validation Data** | Which data was used to compute quantitative metrics? | See validation data. |
| **Simulation Results** | What are the main observations or conclusions mentioned inside the paper regarding the accuracy of the simulation? | Describe the main similarities and discrepancies with the experimental data, as well as any shortcomings of the simulation that are identified.<br><br>For papers without a validation aspect, the main conclusions regarding the simulated motion can be discussed here. |

## Software Details

| | | |
|---|---|---|
| **MSK/Physics Software** | Which (musculoskeletal) physics engine is used to run the simulations? | • DART<br>• SD/FAST<br>• OpenSim<br>• MuJoCo<br>• AnyBody<br>• Custom: paper creates own engine.<br>• Open Dynamics Engine<br>• Simscape Multibody<br>• SCONE (OpenSim)<br>• Unknown: not mentioned in paper.<br>• NA (Not Applicable): e.g. paper using MATLAB to conduct simulations without any engine. |
| **Programming Languages** | Which programming language is used to code the simulations? | The most popular ones include C, C++, Python & MATLAB.<br><br>Multiple options can be included using the separation character ';'. |
| **Programming Libraries** | Does the paper use any popular libraries to achieve the motion prediction? | Options include: OpenAI Gym, OpenSim Moco, IPOPT, SNOPT,CasADi, OpenSim-RL etc. If none, add 'NA'.<br><br>Multiple libraries can be included using the separation character ';'. |
| **Simulation Time** | How much time is required to run the simulation? | Number & description. |
| **Code Available** | Does the paper make available the integral code used to run the simulations? In other words, can the results be reproduced/re-used? | • Yes<br>• No |

## Motion

| | | |
|---|---|---|
| **Movement** | Which motion does the paper simulate? | Specify any type of motion simulated. The most popular ones include gait, jumping, running, sit-to-stand etc.<br><br>Multiple options can be included using the separation character ';'. |
| **Movement Type** | Is the movement normative or not? | Specify whether the motion is normative, pathological (e.g. muscle weakness, diseases), assisted (exoskeletons, orthoses), or amputee (prosthesis).<br><br>Multiple options can be included using the separation character ';'. |

## Model

| | | |
|---|---|---|
| **Model Type** | Which age-group is being simulated? | • Child<br>• Adult<br>• Elder<br>• Other: non-human characters<br><br>Multiple options can be included using the separation character ';'. |
| **Subject Sex** | What is the sex of the subjects used during the experiments? | • Male<br>• Female<br>• Both: male & female<br>• Unspecified<br>• NA: if no validation with subjects is performed |
| **Sex-specific** | Is a distinction made between male & female models and their respective physiology? | • Yes<br>• No |

| | | |
|---|---|---|
| **Subject-specific** | Is the model adapted to the physiology of the subject? | • Yes<br>• No |
| **Personalization Properties** | If the model is adapted to the subject, what element is being personalized? | Popular options include:<br>• scaling: adapting height/mass scaling.<br>• muscle properties: adapting muscle properties.<br>• contact spheres: adapting ground-contact models.<br>• NA: no personalization included.<br><br>Multiple options can be included using the separation character ‘;’. |
| **Model Name** | What is the name of the model used during the simulations? | Popular options include: gait2392, NEURIPS20XX etc. These often refer to the authors of a paper/creators of the model and its date.<br>When a custom model is used, specify ‘custom’. |
| **Model Dimension** | Is movement outside of the sagittal plane possible? | • 2D: No.<br>• 3D: Yes.<br>• unknown: Not specified or clear from the paper. |
| **Ground Contact Model** | Which model is used to represent the ground contact? | Popular options include the Hunt-Crossley contact model. |
| **Model DOF** | How many degrees of freedom does the model contain? | Specify ‘unknown’ if unclear from paper or not specified. |
| **Muscle Model** | Which model is used to represent the muscles? | Most popular one is the Hill-type model. |
| **Model Nmb Muscles** | How many muscles does the model contain?<br><br>*Papers using models with no muscles are excluded from this review.* | Specify ‘unknown’ if unclear from paper or not specified.<br><br>If the paper uses a torque-driven model or no muscles, specify ‘0’. |
| **Other** | | |
| **Comments** | Which questions remain unanswered?<br>Are there other comments about the paper’s content? | |
| **Links** | Interesting links to the paper’s supplementary material, e.g. code or data used. | |

# Bibliography


1. Llobera J, Charbonnier C (2023) Physics-based character animation and human motor control. Physics of Life Reviews 46:190–219. https://doi.org/10.1016/j.plrev.2023.06.012

2. Song S, Kidziński Ł, Peng XB, et al (2021) Deep reinforcement learning for modeling human locomotion control in neuromechanical simulation. J NeuroEngineering Rehabil 18:126. https://doi.org/10.1186/s12984-021-00919-y

3. Kwiatkowski A, Alvarado E, Kalogeiton V, et al (2022) A Survey on Reinforcement Learning Methods in Character Animation. Computer Graphics Forum 41:613–639. https://doi.org/10.1111/cgf.14504

4. Kidziński Ł, Mohanty SP, Ong C, et al (2018) Learning to Run challenge: Synthesizing physiologically accurate motion using deep reinforcement learning

5. De Groote F, Falisse A (2021) Perspective on musculoskeletal modelling and predictive simulations of human movement to assess the neuromechanics of gait. Proc R Soc B 288:20202432. https://doi.org/10.1098/rspb.2020.2432

6. Alamdari A, Krovi VN (2017) A Review of Computational Musculoskeletal Analysis of Human Lower Extremities. In: Human Modelling for Bio-Inspired Robotics. Elsevier, pp 37–73

7. Shanbhag J, Wolf A, Wechsler I, et al (2023) Methods for integrating postural control into biomechanical human simulations: a systematic review. J NeuroEngineering Rehabil 20:111. https://doi.org/10.1186/s12984-023-01235-3

8. Ezati M, Ghannadi B, McPhee J (2019) A review of simulation methods for human movement dynamics with emphasis on gait. Multibody Syst Dyn 47:265–292. https://doi.org/10.1007/s11044-019-09685-1

9. Febrer-Nafría M, Nasr A, Ezati M, et al (2023) Predictive multibody dynamic simulation of human neuromusculoskeletal systems: a review. Multibody Syst Dyn 58:299–339. https://doi.org/10.1007/s11044-022-09852-x

10. Xiang Y, Arora JS, Abdel-Malek K (2010) Physics-based modeling and simulation of human walking: a review of optimization-based and other approaches. Struct Multidisc Optim 42:1–23. https://doi.org/10.1007/s00158-010-0496-8

11. Firouzi V, Seyfarth A, Song S, et al (2023) Biomechanical models in the lower-limb exoskeletons development: A review

12. Bersani A, Davico G, Viceconti M (2023) Modeling Human Suboptimal Control: A Review. Journal of Applied Biomechanics 39:294–303. https://doi.org/10.1123/jab.2023-0015

13. Mahdian ZS, Wang H, Irfan Mohamed Refai M, et al (2023) Tapping into skeletal muscle biomechanics for design and control of lower-limb exoskeletons: a narrative review

14. Lund ME, De Zee M, Andersen MS, Rasmussen J (2012) On validation of multibody musculoskeletal models. Proc Inst Mech Eng H 226:82–94. https://doi.org/10.1177/0954411911431516

15. Mueller-Alexander J LibGuides: Engineering (Basic): Formulating questions w/PICO. https://perma.cc/E8UG-KXZC. Accessed 18 Jul 2022
