# Supplementary material for: A PRISMA systematic review through time on predictive musculoskeletal simulations
Source: J Neuroeng Rehabil. 2025 Jul 4;22:149. doi: 10.1186/s12984-025-01686-w (PMC12228224; doi:10.1186/s12984-025-01686-w)
Supplement: Supplementary file 3 — Supplementary Material 3. This file summarizes additional results from the review paper, including a table of metrics for each method. [file 12984_2025_1686_MOESM3_ESM.pdf]

A PRISMA Systematic Review Through Time on Predictive Musculoskeletal Simulations

Supplementary Material

Menthy Denayer, Eligia Alfio, María Alejandra Díaz, Massimo Sartori, Friedl De Groote, Kevin De Pauw and Tom Verstraten

This document contains additional information and results from the PRISMA systematic review on predictive musculoskeletal simulations. The supplementary material contains three parts:

- **Supplementary Material on Results**: this document gives additional information on the methodology, inter-rater agreement metrics and the quantitative validation of predictive simulations.
- **PRISMA Scoping Review Details**: this document details the process of conducting the review, the criteria, used software and data entries.
- **Data Collection**: This document contains the extracted data entries for the screened papers.

Contents

1 Methodology **2**
1.1 Search Strategy . . . 2
1.2 Inter-Rater Agreement Metrics . . . 2
1.3 Inclusion & Exclusion Criteria . . . 2
1.4 Data Entries . . . 2

2 Results **4**
2.1 Experimental Data . . . 4
2.2 Quantitative Validation . . . 4

1 Methodology

1.1 Search Strategy

During the initial title screening, we excluded 746 papers. Of these, 31% focus on the analysis of data, sensor measurements, in vivo experiments, imaging or stiffness estimations. Additionally, 11% dealt with out-of-scope topics, like drones, energetic models or virtual reality environments, and around 9% with robot design and control.

During the abstract screening we excluded 423 papers. Of these papers, 34% presented different types of simulations (modelling, analysis etc.) or no simulation at all, while 28% dealt with motions excluded from the scope of the review (Section 1.3), and 20% of papers used only data to actuate the model, thus not fitting our definition of predictive motion. Finally, 10% of papers used torque-driven, character or animal models and 8% of papers were reviews.

1.2 Inter-Rater Agreement Metrics

The inter-rater agreement metrics are computed using the following formulas. For the Cohen's Kappa (κ) [1]:

$$\kappa = \frac{\pi_o - \pi_e}{1 - \pi_e}, \quad (1)$$

$$\pi_o = \frac{N - N_{conflicts}}{N}, \quad (2)$$

$$\pi_e = \frac{1}{N^2}(n_{incl,1}n_{incl,2} + n_{excl,1}n_{excl,2}), \quad (3)$$

with N the total number of screened papers, $N_{conflicts}$ the number of conflicting decisions, and $n_{incl,i}$, $n_{excl,i}$ the number of included and excluded papers for reviewer i.

For the intra-class correlation coefficient (r) [2]:

$$r = \frac{1}{Ns^2}\sum_{j=1}^{N}(x_{j,1} - \bar{x})(x_{j,2} - \bar{x}), \quad (4)$$

$$\bar{x} = \frac{1}{2N}\sum_{j=1}^{N}(x_{j,1} + x_{j,2}), \quad (5)$$

$$s^2 = \frac{1}{2N}\left(\sum_{j=1}^{N}(x_{j,1} - \bar{x})^2 + \sum_{j=1}^{N}(x_{j,2} - \bar{x})^2\right), \quad (6)$$

with $x_{j,i}$ the decision (1: included, 0: excluded) by reviewer i for paper j.

1.3 Inclusion & Exclusion Criteria

A detailed list of criteria at every stage of the review process is added in the "PRISMA Scoping Review Details" document.

1.4 Data Entries

A detailed list of data entries can be found in the "PRISMA Scoping Review Details" document. We extracted data related to the publication (year, journal, Q-value, research group or institute), the methodology, software, generated motion, and the used model.

For the methodology, we determined whether the studies modelled the neural controller (e.g. muscle-reflex-based) or used optimization to make up for the lack of such a model (objective optimization). We also collected the method of achieving the motion (e.g. optimal control, reinforcement learning etc.) and the main algorithm, model or controller. Additionally, we collected the optimization variables and described the cost function for the papers using optimization. We defined papers using experimental data as using imitation learning. Additionally, we classified whether papers could generate motion independent of experimental data (fully predictive) or not (semi-predictive). Papers using measured data to tune model parameters, where the same controller can be used afterwards in a new situation, are also considered fully predictive. Finally, we determined whether the motion was validated, and, if so, which validation data was employed and how many subjects were used.

For the software, we extracted the MSK or physics engine, the programming languages and libraries, the simulation time and whether the code is publicly available.

We extracted the generated motion (e.g. walking) and whether it is normative (healthy), pathological, perturbed, loaded (i.e. while carrying loads), assisted by an external device or representing an individual with an amputation.

Finally, we determined whether the model represented a child, adult or older adult, the sex of the subjects used for validation and whether the model was adapted to the subject's sex or their anatomical properties. For personalized models, we extracted personalized model properties. Finally, we extracted the model's name, the ground contact model, the muscle model, the model dimension (restrained to the sagittal plane or not), the number of degrees of freedom (DOF) and muscles.

2 Results

2.1 Experimental Data

To contextualize the computed metrics of the simulations, we analyzed the data by Van Criekinge et al. [3] (Table 1). We computed the root mean square (RMS) value of 2σ to get an idea of inter-and intra-subject differences. Additionally, we compute Pearson correlation coefficients, for each subject, between individual measurements. We repeated the same for the means of each subject, computing the correlations between subject means and the RMS value for the inter-subject standard deviation.

$$\mathrm{RMS}_x^{\mathrm{inter}} = \mathrm{rms}(2\sigma_x^{\mathrm{inter}}) \tag{7}$$

$$\mathrm{RMS}_x^{\mathrm{intra}} = \mathrm{rms}(2\sum_s \sigma_{x,s}^{\mathrm{intra}}), \tag{8}$$

where x represents one of the six variables and $\sum_s$ the mean over all subjects.

$$\mathrm{R}_x^{\mathrm{inter}} = \sum_{i \neq j} \mathcal{P}(\bar{x}_i, \bar{x}_j)\ \{i, j = 1...N_s\} \tag{9}$$

$$\mathrm{R}_x^{\mathrm{intra}} = \sum_s \sum_{k \neq l} \mathcal{P}(x_k^s, x_l^s)\ \{k, l = 1...N_{it}^s\}, \tag{10}$$

where $\mathcal{P}$ computes the Pearson correlation between two measurements, $\bar{x}_i$ denotes the mean measurement for subject i, x_k^s the measurement for subject s and run k, N_s the number of subjects and N_{it}^s the total number of experiment iterations for subject s and Σ the mean operator.

Table 1: Metrics computed for walking, based on the data by Van Criekinge et al. [3], between subjects (inter) and between multiple measurements of one subject (intra).

**type**	**metric**	**joint angles**			**ground reaction forces**		
		hip	knee	ankle	x (horz.)	y (horz.)	z (vert.)
intra	RMS	3.46°	5.18°	3.32°	0.27 N/kg	0.13 N/kg	1.97 N/kg
	R	0.99	0.98	0.94	0.82	0.71	0.75
inter	RMS	16.82°	12.86°	10.07°	0.53 N/kg	0.23 N/kg	3.03 N/kg
	R	0.99	0.97	0.87	0.86	0.72	0.82

2.2 Quantitative Validation

For OC solutions, Falisse et al. [4] achieve a RMSE below 10° for the stance knee and ankle angle, when modelling the toe joint. They even reduce the stance ankle joint angle to 6.3° by reducing the Achilles tendon stiffness by 60%. Millard et al. [5] report maximal RMSEs of 20.7°, 17.7° and 9.6°, when using a double circle ground contact model. Miller et al. [6] simulate sprinting and reach RMSEs - reported as a number of standard deviations compared to experimental data - for the hip, knee and ankle angle of 0.9SD, 0.8SD and 0.8SD, respectively, when minimizing the square of muscle activations. Minimizing the cost of transport and muscle stress lead to RMSEs above 1SD for one or more joints. The GRFs show RMSEs above 2.7SD for all cost functions. The vertical GRF RMSE was lowest when minimizing the muscle activation squared and the horizontal GRF RMSE when minimizing the muscle stress. Miller et al. [6] also report a correlation coefficient between EMG measurements and the simulated muscle activations of 0.67 ± 0.36 when minimizing the muscle activations squared. The highest correlation appears for the plantarflexors (0.94) and the lowest for the rectus femoris (0.16). The above papers do not use experimental data when predicting movement, except to create an initial guess for the simulation.

Halloran et al. [7], using tracking terms achieve RMSE below 2° for the hip, knee and ankle joint angles by minimizing fatigue and tracking experimental data. Nguyen et al. [8] reach a total RMSE (for both the kinematics and GRFs) of 1.81 after applying bilevel optimization. The same optimization, minimizing the cubed muscle activations results in a total RMSE of 5.39.

The muscle-reflex solution of Geyer & Herr [9] reaches correlation values between measured EMG signals and the simulated muscle activations, during stance, of 0.97 (SOL), 0.99 (GAS), 0.93 (GLU), 0.90 (HAM), 0.87 (VAS), 0.87 (TA) and 0.84 (HFL), and during swing, 0.95 (HAM), 0.87 (HFL), 0.87 (TA), 0.51 (GLU), 0.51 (VAS). Other works, using optimization to tune the muscle-reflex model parameters, reach similar values for the joint angles. Jin et al. [10] reach correlations of 0.72 (FEM), 0.70 (HAM), 0.97 (GAS), 0.93 (SOL) and 0.15 (TA) for the muscle excitations, at a speed of 1.1 m/s. Finally, Song et al. [11] report correlation values of 0.73 (HAB), 0.32 (HAD), 0.86 (HFL), 0.89 (GLU), 0.49 (HAM), 0.76 (RF), 0.85 (VAS), 0.82 (BFSH), 0.97 (GAS), 0.90 (SOL) and 0.81 (TA) for the muscles.

Table 2: Summary of reported metrics for walking for optimal control (OC), deep reinforcement learning (DRL), muscle-reflex-based models (reflex) and central pattern generators (CPG). Metrics are given for the ankle (A), knee (K), hip (H), horizontal (H) or vertical (V) ground reaction forces, for stance (St) or swing (Sw). Red denotes works using experimental data (for OC & DRL), while blue works use optimization to model reflex or CPG parameters. (ND: no available data)

**metric**	**OC**				**reflex**				**DRL**				**CPG**			
								joint angles								
	ref	A	K	H	ref	A	K	H	ref	A	K	H	ref	A	K	H
RMSE	[5]	4.2°	7.5°	12.8°	[17]	0.82 SD	1.48 SD	1.55 SD	[14]	2.59°			ND			
	[18]	2.3°	3.2°	3.1°	[19]	0.99 SD	1.99 SD	1.28 SD	[20]	≤ 2.20 SD						
	$[4]_{St}$	8.2°	5.3°	ND												
	$[21]_{St}$	4.15 SD	2.56 SD	2.52 SD												
	$[21]_{Sw}$	3.76 SD	5.34 SD	4.58 SD												
	[7]	1.9°	1.5°	1.5°												
R	$[21]_{St}$	0.91	0.99	0.99	[22]	0.93	1.00	0.98	[13]	0.09	0.96	0.97	[16]	0.31	0.81	0.89
	$[21]_{Sw}$	0.80	0.97	0.96	$[9]_{St}$	0.96	0.97	0.98	[12]	> 0.92			[15]	0.74	0.95	0.98
	[18]	0.99			$[9]_{Sw}$	0.63	0.89	0.99	[23]	0.82 < R < 0.98						
					[24]	0.22	0.89	0.85								
					[10]	0.65	0.88	0.92								
					[11]	0.46	0.97	0.97								
					[19]	0.89	0.98	0.95								
					[25]	-0.10	0.97	0.98								
								joint torques								
RMSE	[4]	10.6 Nm	6.2 Nm	ND	[17]	1.17 SD	1.46 SD	1.08 SD	ND				ND			
	$[21]_{St}$	11.28 SD	7.52 SD	5.95 SD	[19]	2.56 SD	1.92 SD	1.63 SD								
	$[21]_{Sw}$	13.93 SD	16.78 SD	11.75 SD												
R	$[21]_{St}$	0.97	0.86	0.93	[22]	1.00	0.93	0.80	ND				[16]	0.88	0.30	0.60
	$[21]_{Sw}$	0.82	0.86	0.84	[9]	0.99	0.65	0.45								
					[11]	0.90	0.50	0.87								
					[19]	0.96	0.87	0.90								
								ground reaction forces								
	ref	H	V		ref	H	V		ref	H	V		ref	H	V	
RMSE	[5]	40.2 N	105.4 N		$[17]_{St}$	1.04 SD	0.78 SD		[20]	≤ 0.55 BW						
	[18]	9.9 N	27.2 N		[19]	4.09 SD	2.29 SD									
R	[18]	0.97	0.99		[24]	0.80	0.96		[23]	ND	0.98		[16]	0.86	ND	
					[11]	0.67	0.94						[15]	0.46	0.97	
					[19]	0.92	0.99									
					[25]	0.27	0.55									

Only 2 papers report quantitative metrics for DRL solutions when no experimental data is used. Weng et al. [12] reach a mean correlation higher than 0.92 for the hip, knee and ankle joint angles. Su et al. [13], combining muscle-reflex models and DRL, report correlations above 0.96 for the hip and knee joint angles, but only 0.09 for the ankle joint angle. Qin et al. [14] report an average RMSE of 2.59°, 8.95° and 3.62° for walking, running and jumping, respectively.

Ichimura et al. [15] are the only one reporting quantitative metrics when using only a CPG model. They compute cosine similarity values of 0.72 (GM), 0.53 (RF), 0.68 (VA), 0.66 (BFL), 0.74 (TA), 0.84 (SO), 0.30 (GC) for the muscles compared to EMG data. Aoi et al. [16] combine a CPG model with muscle synergies. For walking, they reach correlations above 0.81 for the hip and knee joint angles, but only 0.31 for the ankle joint angles. For running, the correlations are higher for the joint angles: 0.91 (hip), 0.89 (knee), 0.57 (ankle) and for the joint torques 0.45 (hip), 0.87 (knee), 0.92 (ankle). For the GRFs the correlations are 0.86 (walk) and 0.42 (run) for the horizontal component and the cosine similarity is 0.92 (walk) and 0.80 (run) for the vertical component. Aoi et al. [16] also compute the cosine similarity for the muscles during walking: 0.60 (IL), 0.78 (GM), 0.58 (VA), 0.57 (BFS), 0.44 (TA), 0.86 (SO), 0.68 (RF), 0.60 (BFL), 0.71 (GC) and running: 0.66 (IL), 0.86 (GM), 0.88 (VA), 0.48 (BFS), 0.77 (TA), 0.91 (SO), 0.65 (RF), 0.52 (BFL), 0.81 (GC).

References

1. Landis, J. R. & Koch, G. G. The Measurement of Observer Agreement for Categorical Data. en. *Biometrics* **33,** 159. ISSN: 0006341X. https://www.jstor.org/stable/2529310?origin=crossref (2024) (Mar. 1977).
2. Bartko, J. J. The Intraclass Correlation Coefficient as a Measure of Reliability. en. *Psychol Rep* **19,** 3–11. ISSN: 0033-2941, 1558-691X. https://journals.sagepub.com/doi/10.2466/pr0.1966.19.1.3 (2024) (Aug. 1966).
3. Van Criekinge, T. *et al.* A full-body motion capture gait dataset of 138 able-bodied adults across the life span and 50 stroke survivors. en. *Sci Data* **10,** 852. ISSN: 2052-4463. https://www.nature.com/articles/s41597-023-02767-y (2025) (Dec. 2023).
4. Falisse, A., Afschrift, M. & De Groote, F. Modeling toes contributes to realistic stance knee mechanics in three-dimensional predictive simulations of walking. *PLOS ONE* **17.** ISSN: 1932-6203. (2022) (Jan. 2022).
5. Millard, M. & Mombaur, K. A Quick Turn of Foot: Rigid Foot-Ground Contact Models for Human Motion Prediction. en. *Front. Neurorobot.* **13,** 62. ISSN: 1662-5218. https://www.frontiersin.org/article/10.3389/fnbot.2019.00062/full (2024) (Aug. 2019).
6. Miller, R. H., Umberger, B. R., Hamill, J. & Caldwell, G. E. Evaluation of the minimum energy hypothesis and other potential optimality criteria for human running. *Proceedings of the Royal Society B: Biological Sciences* **279,** 1498–1505. ISSN: 0962-8452. (2012) (Apr. 2012).
7. Halloran, J. P., Ackermann, M., Erdemir, A. & Van Den Bogert, A. J. Concurrent musculoskeletal dynamics and finite element analysis predicts altered gait patterns to reduce foot tissue loading. en. *J. Biomech.* **43,** 2810–2815. ISSN: 00219290. https://linkinghub.elsevier.com/retrieve/pii/S0021929010003234 (2024) (Oct. 2010).
8. Nguyen, V. Q., Johnson, R. T., Sup, F. C. & Umberger, B. R. Bilevel Optimization for Cost Function Determination in Dynamic Simulation of Human Gait. en. *IEEE Trans. Neural Syst. Rehabil. Eng.* **27,** 1426–1435. ISSN: 1534-4320, 1558-0210. https://ieeexplore.ieee.org/document/8736354/ (2024) (July 2019).
9. Geyer, H. & Herr, H. A Muscle-Reflex Model That Encodes Principles of Legged Mechanics Produces Human Walking Dynamics and Muscle Activities. en. *IEEE Trans. Neural Syst. Rehabil. Eng.* **18,** 263–273. ISSN: 1534-4320, 1558-0210. https://ieeexplore.ieee.org/document/5445011/ (2024) (June 2010).
10. Jin, W. *et al.* Forward dynamics simulation of a simplified neuromuscular-skeletal-exoskeletal model based on the CMA-ES optimization algorithm: framework and case studies. en. *Multibody Syst Dyn* **62,** 525–558. ISSN: 1384-5640, 1573-272X. https://link.springer.com/10.1007/s11044-024-09982-4 (2025) (Dec. 2024).
11. Song, S. & Geyer, H. A neural circuitry that emphasizes spinal feedback generates diverse behaviours of human locomotion. en. *J. Physiol.* **593,** 3493–3511. ISSN: 0022-3751, 1469-7793. https://physoc.onlinelibrary.wiley.com/doi/10.1113/JP270228 (2024) (Aug. 2015).
12. Weng, J., Hashemi, E. & Arami, A. Natural Walking With Musculoskeletal Models Using Deep Reinforcement Learning. en. *IEEE Robot. Autom. Lett.* **6,** 4156–4162. ISSN: 2377-3766, 2377-3774. https://ieeexplore.ieee.org/document/9382086/ (2024) (Apr. 2021).
13. Su, B. & Gutierrez-Farewik, E. M. Simulating human walking: a model-based reinforcement learning approach with musculoskeletal modeling. en. *Front. Neurorobot.* **17,** 1244417. ISSN: 1662-5218. https://www.frontiersin.org/articles/10.3389/fnbot.2023.1244417/full (2024) (Oct. 2023).
14. Qin, W., Tao, R., Sun, L. & Dong, K. Muscle-driven virtual human motion generation approach based on deep reinforcement learning. en. *Computer Animation & Virtual* **33,** e2092. ISSN: 1546-4261, 1546-427X. https://onlinelibrary.wiley.com/doi/10.1002/cav.2092 (2024) (June 2022).
15. Ichimura, D., Hobara, H., Hisano, G., Maruyama, T. & Tada, M. Acquisition of bipedal locomotion in a neuromusculoskeletal model with unilateral transtibial amputation. *Frontiers in Bioengineering and Biotechnology* **11.** ISSN: 2296-4185. (2023) (Mar. 2023).
16. Aoi, S. *et al.* Neuromusculoskeletal model that walks and runs across a speed range with a few motor control parameter changes based on the muscle synergy hypothesis. *Scientific Reports* **9.** Univ Electrocommun. ISSN: 2045-2322. (2019) (Jan. 2019).
17. Ong, C. F., Geijtenbeek, T., Hicks, J. L. & Delp, S. L. Predicting gait adaptations due to ankle plantarflexor muscle weakness and contracture using physics-based musculoskeletal simulations. en. *PLoS Comput Biol* **15** (ed Srinivasan, M.) e1006993. ISSN: 1553-7358. https://dx.plos.org/10.1371/journal.pcbi.1006993 (2024) (Oct. 2019).
18. Weng, J., Hashemi, E. & Arami, A. Human Gait Cost Function Varies With Walking Speed: An Inverse Optimal Control Study. *IEEE Robot. Autom. Lett.* **8,** 4777–4784. ISSN: 2377-3766. (2023) (Aug. 2023).
19. Waterval, N. *et al.* Validation of forward simulations to predict the effects of bilateral plantarflexor weakness on gait. en. *Gait & Posture* **87,** 33–42. ISSN: 09666362. https://linkinghub.elsevier.com/retrieve/pii/S0966636221001417 (2024) (June 2021).
20. De Vree, L. & Carloni, R. Deep Reinforcement Learning for Physics-Based Musculoskeletal Simulations of Healthy Subjects and Transfemoral Prostheses' Users During Normal Walking. en. *IEEE Trans. Neural Syst. Rehabil. Eng.* **29,** 607–618. ISSN: 1534-4320, 1558-0210. https://ieeexplore.ieee.org/document/9366532/ (2024) (2021).
21. D'Hondt, L., De Groote, F. & Afschrift, M. A dynamic foot model for predictive simulations of human gait reveals causal relations between foot structure and whole-body mechanics. en. *PLoS Comput Biol* **20** (ed Haith, A. M.) e1012219. ISSN: 1553-7358. https://dx.plos.org/10.1371/journal.pcbi.1012219 (2025) (June 2024).

22. Dzeladini, F., Van Den Kieboom, J. & Ijspeert, A. The contribution of a central pattern generator in a reflex-based neuromuscular model. en. *Front. Hum. Neurosci.* **8.** ISSN: 1662-5161. `http://journal.frontiersin.org/article/10.3389/fnhum.2014.00371/abstract` (2024) (June 2014).
23. Anand, A. S., Zhao, G., Roth, H. & Seyfarth, A. *A deep reinforcement learning based approach towards generating human walking behavior with a neuromuscular model* en. in *2019 IEEE-RAS 19th International Conference on Humanoid Robots (Humanoids)* (IEEE, Toronto, ON, Canada, Oct. 2019), 537–543. ISBN: 978-1-5386-7630-1. `https://ieeexplore.ieee.org/document/9035034/` (2024).
24. He, H., Li, K., Zhang, J., Xie, S. & Zhang, Z.-Q. *Modifying Gait: A Strategy for Joint Reaction Forces Reduction in Below-Knee Amputees* en. in *2023 29th International Conference on Mechatronics and Machine Vision in Practice (M2VIP)* (IEEE, Queenstown, New Zealand, Nov. 2023), 1–6. ISBN: 9798350325621. `https://ieeexplore.ieee.org/document/10413436/` (2024).
25. Koseki, S., Hayashibe, M. & Owaki, D. Identifying essential factors for energy-efficient walking control across a wide range of velocities in reflex-based musculoskeletal systems. en. *PLoS Comput Biol* **20** (ed Liu, J.) e1011771. ISSN: 1553-7358. `https://dx.plos.org/10.1371/journal.pcbi.1011771` (2025) (Jan. 2024).
